# Intelligent agriculture: deep learning in UAV-based remote sensing imagery for crop diseases and pests detection

**DOI:** 10.3389/fpls.2024.1435016

**Published:** 2024-10-24

**Authors:** Hongyan Zhu, Chengzhi Lin, Gengqi Liu, Dani Wang, Shuai Qin, Anjie Li, Jun-Li Xu, Yong He

**Affiliations:** ^1^ Guangxi Key Laboratory of Brain-inspired Computing and Intelligent Chips, School of Electronic and Information Engineering, Guangxi Normal University, Guilin, China; ^2^ Key Laboratory of Integrated Circuits and Microsystems (Guangxi Normal University), Education Department of Guangxi Zhuang Autonomous Region, Guilin, China; ^3^ School of Biosystems and Food Engineering, University College Dublin, Dublin, Ireland; ^4^ College of Biosystems Engineering and Food Science, Zhejiang University, Hangzhou, China

**Keywords:** intelligent agriculture (IA), deep learning (DL), crop diseases and pests, remote sensing (RS), unmanned aerial vehicle (UAV)

## Abstract

Controlling crop diseases and pests is essential for intelligent agriculture (IA) due to the significant reduction in crop yield and quality caused by these problems. In recent years, the remote sensing (RS) areas has been prevailed over by unmanned aerial vehicle (UAV)-based applications. Herein, by using methods such as keyword co-contribution analysis and author co-occurrence analysis in bibliometrics, we found out the hot-spots of this field. UAV platforms equipped with various types of cameras and other advanced sensors, combined with artificial intelligence (AI) algorithms, especially for deep learning (DL) were reviewed. Acknowledging the critical role of comprehending crop diseases and pests, along with their defining traits, we provided a concise overview as indispensable foundational knowledge. Additionally, some widely used traditional machine learning (ML) algorithms were presented and the performance results were tabulated to form a comparison. Furthermore, we summarized crop diseases and pests monitoring techniques using DL and introduced the application for prediction and classification. Take it a step further, the newest and the most concerned applications of large language model (LLM) and large vision model (LVM) in agriculture were also mentioned herein. At the end of this review, we comprehensively discussed some deficiencies in the existing research and some challenges to be solved, as well as some practical solutions and suggestions in the near future.

## 1 Introduction

Crop diseases and pests are the major natural disasters affecting agricultural production. They are the main factors restricting high yield, high quality, high efficiency, ecology, and agriculture safety. The food and agriculture organization (FAO) of the United Nations reports that pests account for an annual loss of approximately 10-15% in global crop yields, totaling over 300 million tons (Qi et al., 2019). Crop diseases and pests have the characteristics of many kinds, significant influence, and break out easily, which will cause the decline of crop quality and yield. Therefore, in actual agricultural production, the rapid acquisition of crop pest information and early detection of pests and diseases is critical for achieving crop yield increase and reducing disease losses (Thangaraj et al., 2021). It is also an essential basis for the implementation of intelligent agriculture (IA).

Traditional detection methods of crop diseases and pests mainly rely on artificial visual evaluation, serology, and molecular biology-based technical means. Examples of these technical means include flow cytometry, enzyme-linked immunosorbent assay (ELISA), immunofluorescence (IF), polymerase chain reaction (PCR), and fluorescence *in situ* hybridization (FISH) (Carvajal-Yepes et al., 2019). Although these crop disease detection technologies can accurately diagnose crop diseases, they have some drawbacks. These drawbacks include being time-consuming, inefficient, and destructive, requiring detailed sampling and processing procedures, requiring technicians with strong professional knowledge and skills, and being greatly affected by human factors. It is challenging to detect large-scale full-coverage field crop diseases and pests effectively, which limits the development of IA and crop breeding. Therefore, how to quickly, accurately, and efficiently monitor the occurrence of crop diseases and pests in a wide range and timely prevent and control early conditions has become a significant problem in crop production. In addition, how to minimize the losses caused by pests and diseases to crops is also an essential issue that needs to be addressed.

At present, the detection of crop diseases is divided into several types. These types include indoor fine detection based on leaf detection, agricultural machinery as the main platform for a single plant or small-scale crop canopy field fine detection, aircraft monitoring platforms for crop canopy farmland plot scale detection, and satellite images as the data source of regional scale detection (Zhang et al., 2019). Ground-level RS platforms including indoor and field fine detection scales have the characteristics of a controllable environment and high accuracy. However, they cannot obtain crop disease information in an extensive range (Liu et al., 2020). Satellite-level RS has some shortcomings, such as low spatial and temporal resolution, poor timeliness, and low accuracy. The low-altitude RS platform represented by UAVs has become a new means for obtaining crop disease information at the scale of farmland plots under its advantages of flexibility, low cost, and suitability for complex farmland environments (Mukherjee et al., 2019). The UAV platforms can carry a variety of sensors, including RGB cameras, multi-spectral cameras, infrared thermal cameras, hyperspectral cameras, laser radar, etc (Yang et al., 2017). Among these sensors, RGB cameras, multi-spectral cameras, and other spectral imaging sensors are widely used to acquire farmland crop disease information due to their lightweight, low-cost, and easy operation. After diseases attack crops, their color, texture, and spectral characteristics will change to a certain extent, and the effects of different diseases on crops are also disparate. Accordingly, the limitations of traditional detection methods, such as their reliance on manual labor and susceptibility to human error, are significantly addressed by UAV remote sensing technology, which enables rapid, accurate, and scalable monitoring of crop health, thereby fostering new opportunities for intelligent agriculture.

The unmanned aerial systems consist of different integrating sensors (high-resolution RGB, multispectral, hyperspectral, Li-DAR, and thermal), Internet connectivity, flight missions, data collection, image processing, and AI algorithms (García et al., 2020). One of the most common applications is the assessment of crop health through RS and image processing (Radoglou-Grammatikis et al., 2020), which is the focus of this paper. UAV is remotely controlled by an operator and can carry a variety of cameras such as multi-spectral and hyper-spectral, thus acquiring aerial images, providing a wealth of information on crop growth, health status, and environmental conditions. However, the full potential of UAV-collected data can only be realized through sophisticated image processing and analysis techniques (Peñuelas et al., 1995). Deep learning, a powerful branch of machine learning, has demonstrated remarkable capabilities in analyzing and interpreting complex image data. By leveraging deep neural networks, deep learning algorithms can extract meaningful features from raw imagery, enabling precise classification and detection of crop diseases and pests.

This paper focuses on UAV-based hardware devices and imagery process methods that are used in crop diseases and pests. The apparatus comprises standard sensor types, featuring AI-driven processing of UAV RS images employing traditional ML algorithms as well as emerging DL algorithms, to achieve larger scale, faster, and higher accurate surveillance and management of crop diseases and pests. To sum up, we made some contributions:

1. Finding the hottest topics in IA related to ‘UAV’, ‘remote sensing’ and ‘deep learning’. A presentation of keyword co-occurrence analysis, an authors’ co-occurrence analysis, and geographical relations.
2. An explanation of crop diseases and pests detection. A synopsis of typical types of UAVs and sensors. As well as the technology road-map of the RS system.
3. A summary of the common vegetation indices and texture characteristics, application of the wavelength selection algorithms which were widely used in features extraction.
4. A review of some examples in crop detection using traditional ML methods and prominent DL methods in recent years. A brief description of these algorithms and an elaborate discussion of several detailed areas in applying UAV-based RS for crop protection.
5. Proposing some deficiencies in the existing research and challenges to be solved. A prospect of the future of deep learning and artificial general intelligence (AGI) in intelligent agriculture.

## 2 Finding the hot-spots by bibliographic analysis

Using the literature data from the core database of the Web of Science (WOS), we searched the literature on keywords including ‘remote sensing’ and ‘deep learning’ from January 1, 2018, to January 1, 2024, to determine the current international mainstream methods for monitoring crop diseases and pests. To avoid duplication and messy data, we manually cleaned up the papers that did not meet the research theme and finally selected 13738 papers for analysis. Herein, we extracted the abstracts, keywords, authors, countries, journal names, and other information, used VOSviewer (version 1.16.18, Leiden University, Leiden, The Netherlands) to build a database of the extracted data, analyzed the article through clustering, principal component analysis (PCA), and different algorithms to present the results in visual charts (Yang et al., 2020).

### 2.1 Keyword co-occurrence analysis

**Figure 1:**
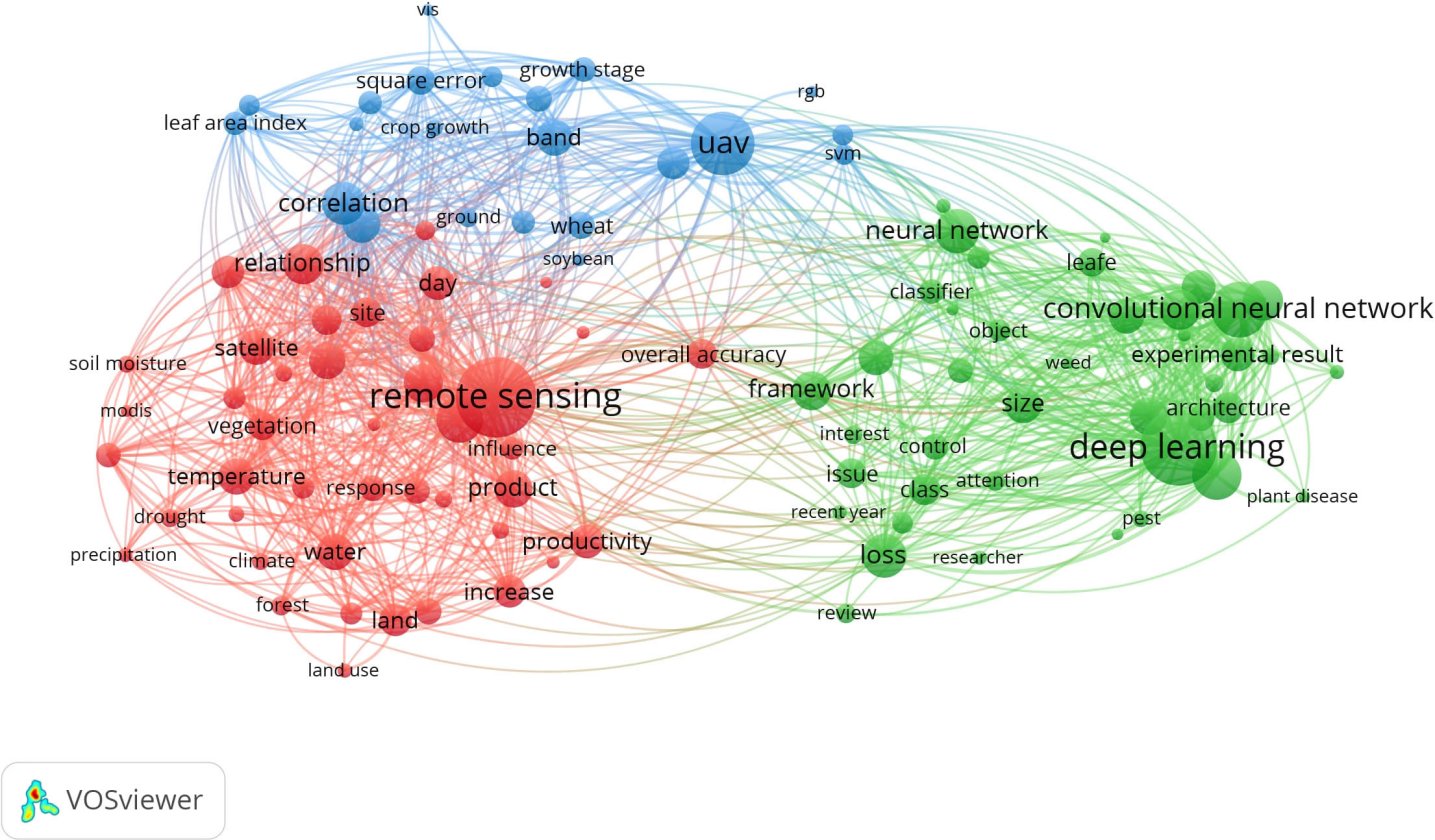
The keyword co-occurrence analysis results.

Firstly, we used the clustering algorithm of VOSviewer to cluster and determine the relationship between the subjects in the selected literature set by the co-occurrence of words or noun phrases in the literature set to obtain the keyword co-occurrence analysis between the core words, including ‘UAV (UAVs/UAS/UAV-based platforms/drones) ‘, ‘remote sensing’ and ‘intelligent agriculture’. As shown in **Figure 1**, the research hot-spots is ‘UAV’, ‘remote sensing’ and ‘deep learning’ from 2018 to 2024. It can be seen that UAV low-altitude RS has a very close cross-connection with IA, especially in crop diseases and pests. The UAV RS system can effectively use vegetation indices to estimate biophysical parameters and generate water stress detection images of leaf area index, chlorophyll content, photochemical reflectance index, and canopy temperature, which is vital for monitoring and prevention of crop diseases and pests (Ren et al., 2020). We clustered the analysis results and used three colors to represent the three clusters. The blue clusters are UAV-centered for correlation analysis, and similarly, the red ones are for remote sensing and the green ones are for deep learning.

### 2.2 Author co-occurrence analysis

**Figure 2:**
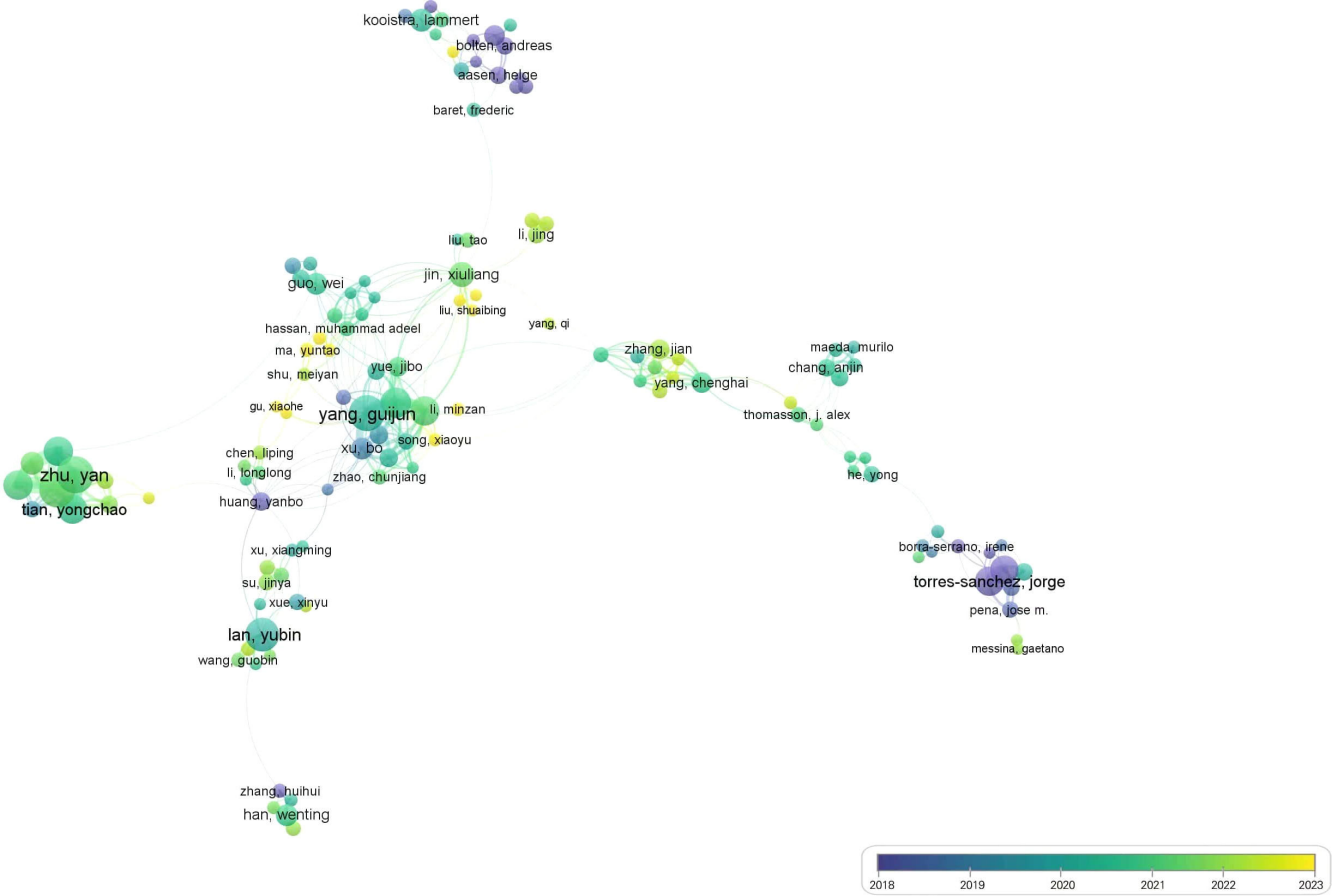
The authors’ co-occurrence analysis results.

Subsequently, we focused on the paper’s authors and the countries for data statistics and analysis. Statistic methods for instance factor analysis, cluster analysis, and PCA were used to judge the research similarity of the literature of two different authors. It is assumed that more than three authors cite the literature of the same two authors, and the citation frequency is high. In that case, it proves that the academic research relationship between the two authors is relatively close. The authors of the selected kinds of literature were classified through VOSviewer. The visualization method showed the academic relationship between the authors who form a scientific community in ‘UAV low-altitude RS ‘ and ‘crop diseases detection’. As illustrated in **Figure 2**, Guijun Yang (the National Engineering Research Center for Information Technology in Agriculture), Yubin Lan (the College of Engineering, South China Agricultural University/National Center for International Collaboration Research on Precision Agricultural Aviation Pesticides Spraying Technology), Yong He (the College of Biosystems Engineering and Food Science, Zhejiang University, Hangzhou), and other authors published many papers on the application of UAV RS in IA from 2018 to 2024 and had close academic relations with many authors.

### 2.3 Analysis of the geographical relationship

**Figure 3:**
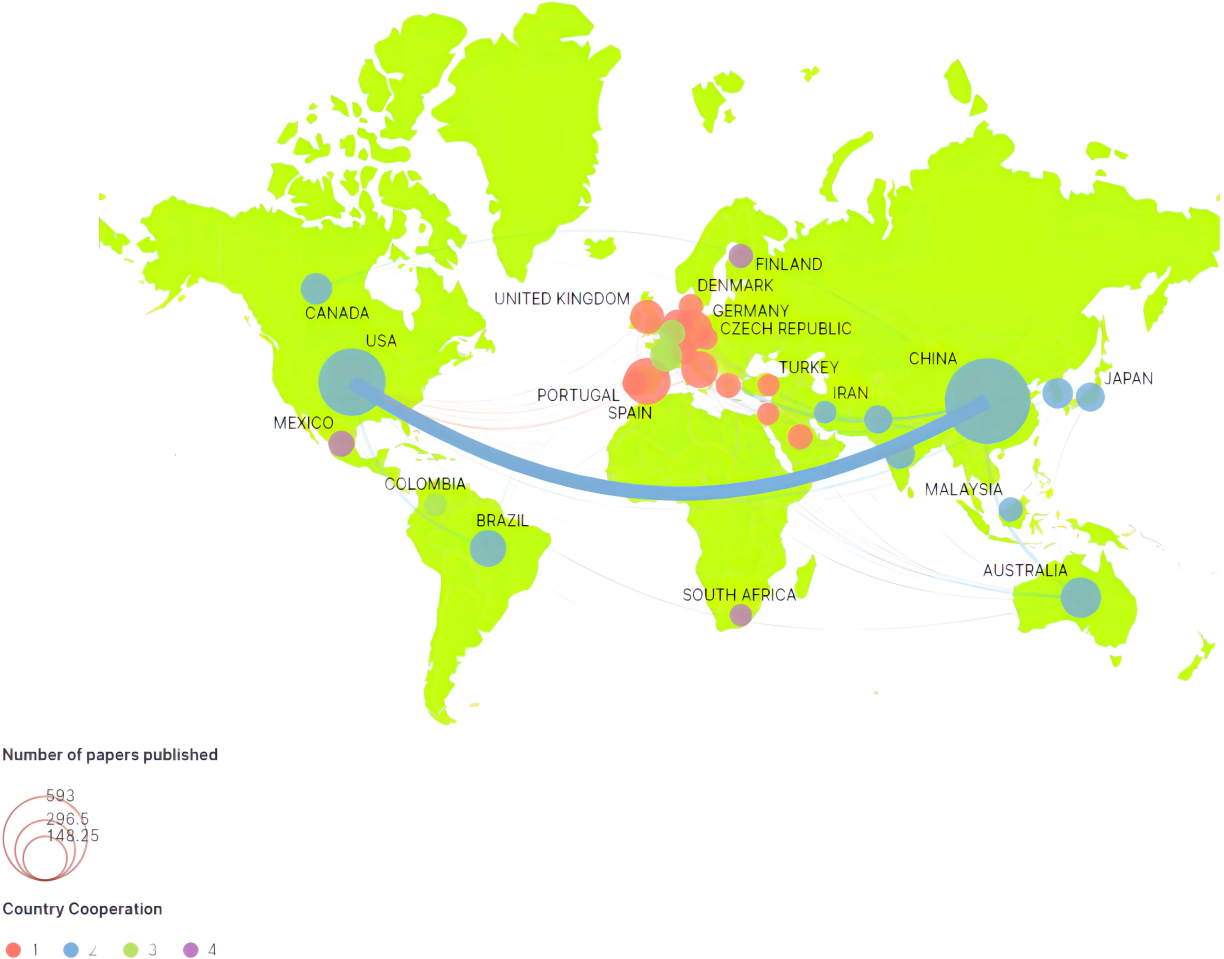
The geographical relations analysis results.

After that, we analyzed the geographical relationship of the authors and used VOSviewer to analyze the degree of academic cooperation among countries. Then we got the national cooperation network map, as shown in **Figure 3**. Moreover, we also statistically analyzed the number of relevant papers published in the past six years. The top ten publishing countries are China, the United States, Australia, Spain, Brazil, Germany, Canada, Italy, the United Kingdom, and Mexico. Two major scientific communities have been formed: a ‘cooperative relationship zone’ dominated by Sino-US cooperation and radiating to Australia, Canada, Brazil, and other countries. The European academic cooperation circle is led by Spain, Germany, Italy, and the United Kingdom and radiates to Turkey, Iran, Israel, and other countries.

### 2.4 Publication and citation of articles on crop diseases and pests monitoring by UAV RS

According to the authors’ co-occurrence analysis, from the co-occurrence map and national cooperation network formed by VOSviewer, low-altitude RS realized by UAVs has gradually become the most powerful tool in recent years (Abd El-Ghany et al., 2020). The application of UAV RS in IA, especially diseases and pest control, has achieved plentiful good results and has gradually become one of the most promising technologies to support integrated pest management (Xu et al., 2022).

The reasons for the wide use of UAVs are obvious. The bacterium, fungi, actinomyces, and other pathogens usually use soil, water, and wind as a medium to spread their spores to make crops sick. The dormancy of adults often causes infestations during overwintering in the previous year and mass breeding in the suitable season of the following year. After diseases and pests attack the crops, their morphological, color, and spectral characteristics (light absorption, reflection, transmission) will change greatly. Different types and degrees of diseases and pests caused by different crop growth conditions, also have more significant differences. Thus, to a certain extent, crop images and spectral information can reflect the occurrence and severity of crop diseases and pests. It also provides a theoretical basis for acquiring and analyzing information about crop diseases and pests by various imaging sensors on the UAV. Many scholars at home and abroad have used this theoretical basis to conduct experimental analysis and published many high-quality articles on crop diseases and pests with the help of UAV RS.

RS based on the UAV platforms has apparent advantages in obtaining pest information of field crops, for instance, high operation efficiency, high spatial-temporal resolution, synchronous image acquisition, and timely field sampling, and excellent structure and texture information. It can carry out rapid qualitative and quantitative research on much information about crop pests, which has been fully reflected in pests monitoring and classification of rice, wheat, corn, and other crops. The advantages of UAV RS provides a real-time and accurate ‘ground-space’ integrated platform for crop diseases and pests monitoring (Ahmad et al., 2022).

## 3 UAV RS in crop diseases and pests

Crop diseases and pests are one of the major agricultural disasters in China, which are characterized by many types, great influence, and frequent outbreaks, and their occurrence and severity often cause significant losses to our national economy, especially agricultural production. The following types of leaf pests and diseases (Zhou et al., 2023) are common: apple scab, black rot, cedar apple rust, rust, grape black rot, and strawberry leaf scorch. From sowing, and growing to harvesting, crops are often victimized by various pests (plant pathogens, pests, weeds and rodents, etc.), thus affecting the yield and quality of cultivated plants, due to the great variety of pests, their different forms, and their different patterns of occurrence. Therefore, it is very difficult to rely only on human experts to recognize pests and master their habits and characteristics. It is extremely important to rely on the methods of ML models and deep learning models to prevent and control pests.

The principles of disease control are: To depress the pathogen or inhibit its occurrence and spread; To improve the disease resistance of the host plant; To control or modify the environmental conditions so that they are favorable to the host plant and unfavorable to the pathogen, and to inhibit the occurrence and development of the disease. Generally, emphasis is placed on the prevention of plant populations, and integrated control measures are taken by the occurrence and development patterns of crop diseases according to location and time.

### 3.1 Definition of pest damage and crop diseases

Pest damage is a phenomenon that harmful insects cause damage to plant growth during the growth of a crop. Crop diseases are the stunting, wilting, or death of a plant body, usually caused by bacteria, fungi, viruses, algae, or unsuitable climate and soil, and is a natural disaster.

Diseases are categorized into two main groups: invasive and non-invasive. The classification of invasive diseases caused by pathogenic organisms is: (1) According to the pathogen is divided into fungal, bacterial, viral, and nematode diseases. (2) According to the host plant is divided into crops, vegetables, fruit tree diseases and forest diseases, etc., but also according to the type of crop is divided into wheat, rice, cotton, and other different crop diseases. (3) According to the symptoms, it can be divided into leaf spot disease, rot disease, wilt disease, and so on. (4) According to the site of disease, it can be divided into root disease, stem disease, leaf disease, fruit disease, and so on. (5) According to the mode of transmission, it can be divided into airborne, waterborne, soil-borne, seedling-borne, insect-mediated transmission, and so on (Chaube and Pundhir, 2005).

### 3.2 Using UAV RS for efficient crop diseases and pests detection

Currently, the detection methods for plant disease mainly include sensory judgment, physical and chemical inspection, conventional machine vision, and other methods, which have high error rates and are easy to cause waste of pesticide spraying and environmental pollution (Zhao et al., 2013). These methods have a high error rate, and for instance, in large areas of tea plantations, disease identification is time-consuming and costly. Therefore, finding a fast and efficient identification method is of great significance to agricultural plant protection.

Crop diseased leaf image segmentation (Huang et al., 2021) is a difficult problem in the research of crop disease recognition methods based on image analysis and computer vision. It is a method that extracts the significant lesion areas of interest from the original lesion leaf images, eliminates the non-significant and unimportant areas, and highlights the important parts of the lesion images, which is conducive to the detection, diagnosis, and identification of crop diseases in the later stage of the disease.

**Figure 4:**
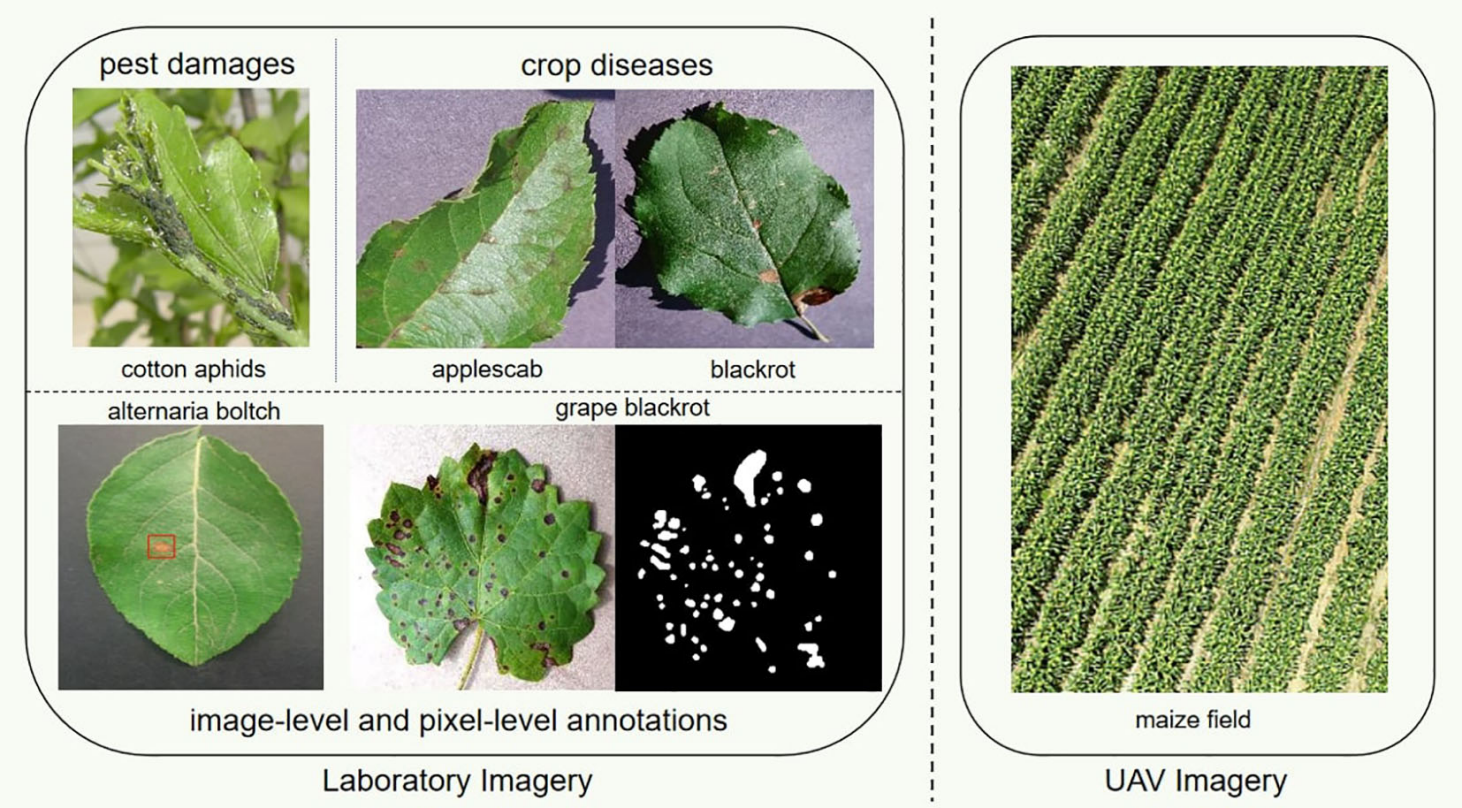
Crop diseases and pests imagery.

Annotation methods play a crucial role in image analysis tasks such as classification, detection, and segmentation. Depending on the specific task, annotation methods can be divided into three categories: bounding box annotations for detection tasks, polygon annotations for segmentation tasks, and categorical labels for classification tasks. Bounding box annotations involve drawing rectangles around objects of interest, while polygon annotations outline the precise shape of objects. Categorical labels, on the other hand, assign a class label to each image or region of an image. By understanding the different types of annotations and how they relate to specific image analysis tasks, researchers can choose the most appropriate annotation method for their particular needs. As shown in **Figure 4**, there is a big difference between the crop images acquired using UAV and the lab images. Most UAV images are canopy images of the plant, containing a variety of data such as leaves, and stalks, and are non-destructive to the crop. Laboratory images require that diseased leaves be removed from the crop and photographed in a laboratory environment. The advantage of laboratory images is their high resolution, which is very useful for pest and disease classification. On the other hand, the resolution of UAV imagery is a big challenge. Imagery with insufficient resolution is difficult to categorize using AI algorithms. Therefore, the choice of the type of cameras and image processing algorithms carried by the UAV is crucial.

### 3.3 Advantages and application prospects

UAV RS technology in IA can quickly cover large areas of farmland, offering a more efficient and flexible approach compared to traditional ground surveys or manned aircraft remote sensing, which has the advantages of monitoring a wide area, in real-time, objective, high efficiency. It not only can effectively reduce the cost of manpower and material resources, but also facilitates a comprehensive grasp of the overall disaster situation, and puts forward more rapid and effective countermeasures, which in turn reduces the damage caused by pests and diseases to crop resources.

Addressing the UAV imagery resolution challenge, the integration of sensors, including high-resolution cameras and multispectral imaging devices, enhances the capabilities of UAVs. This integration empowers UAVs to capture detailed images with superior resolution, enabling precise identification and localization of crop diseases and pests issues, even in their early stages of infestation. Moreover, using multispectral and hyperspectral sensors, UAVs can gather data across different wavelengths. This helps in more accurately analyzing crop health and identifying specific types of pests or diseases. In addition, UAVs can provide timely information on pests and diseases, enabling farmers to take preventive measures and making agricultural management more intelligent. Preventing widespread infestations while reducing the use of insecticides. In summary, UAV remote sensing technology provides an efficient, cost-effective, timely, and accurate method for pest and disease monitoring, which is important for modern agricultural management and crop protection.

## 4 UAV RS systems

The above analysis showed that UAV RS has been widely used in various fields of IA, especially in crop diseases and pests monitoring, and the UAV RS system represented by plant protection UAV has been rapidly developed and applied (He, 2018; Yang K. et al., 2020). A complete set of low-altitude UAV RS systems mainly includes a UAV platform, sensors, a ground station system, and a communication data link. Here, we focus on reviewing the typical types of UAVs, as well as the four typical types of sensors.

### 4.1 Typical types of UAVs

With the rapid development of UAV low-altitude RS, represented by plant protection, UAVs have excellent performance in crop information collection, pest monitoring, spraying, fertilization, and other fields. According to the structure of UAVs, plant protection UAVs are divided into coaxial, single-rotor, and multi-rotor helicopters (Lan et al., 2019). The coaxial plant protection helicopters are generally a hydraulic motor type with solid endurance, sizeable operating area, and other characteristics. But limited by the engine maintenance complexity, as well as the engine life being short and other issues, they are rarely used in practical agricultural operations. Single-rotor plant protection UAVs are mainly the oil-driven type. Compared with coaxial plant protection helicopters, oil-powered single-rotor plant protection drones can effectively reduce maintenance frequency and extend engine life; they have a wide range of crop applications and long working hours. However, because of their high cost and hard to control, they still have certain limitations in large-scale agricultural applications.

Given the limitations of coaxial and single-rotor plant protection drones, electric multi-rotor plant protection drones are widely used in crop disease monitoring. Multi-rotor plant protection UAVs can be divided into four rotors, six rotors, eight rotors, and other unmanned aerial vehicles. The fuselage body is usually made of carbon fiber to reduce its load. With the characteristics of large load, convenient maintenance, sufficient power, etc., they have become the mainstream model of plant protection UAVs (Chen H. et al., 2021; Chen et al., 2017). At present, RS monitoring based on multi-rotor UAVs equipped with visual sensors has been widely used in crop diseases and pests identification (Yinka-Banjo and Ajayi, 2019), growth monitoring, yield estimation (Yang et al., 2015), crop lodging judgment, and other aspects, and RS monitoring provides a new means for crop growth monitoring.

### 4.2 Typical types of sensors

**Figure 5:**
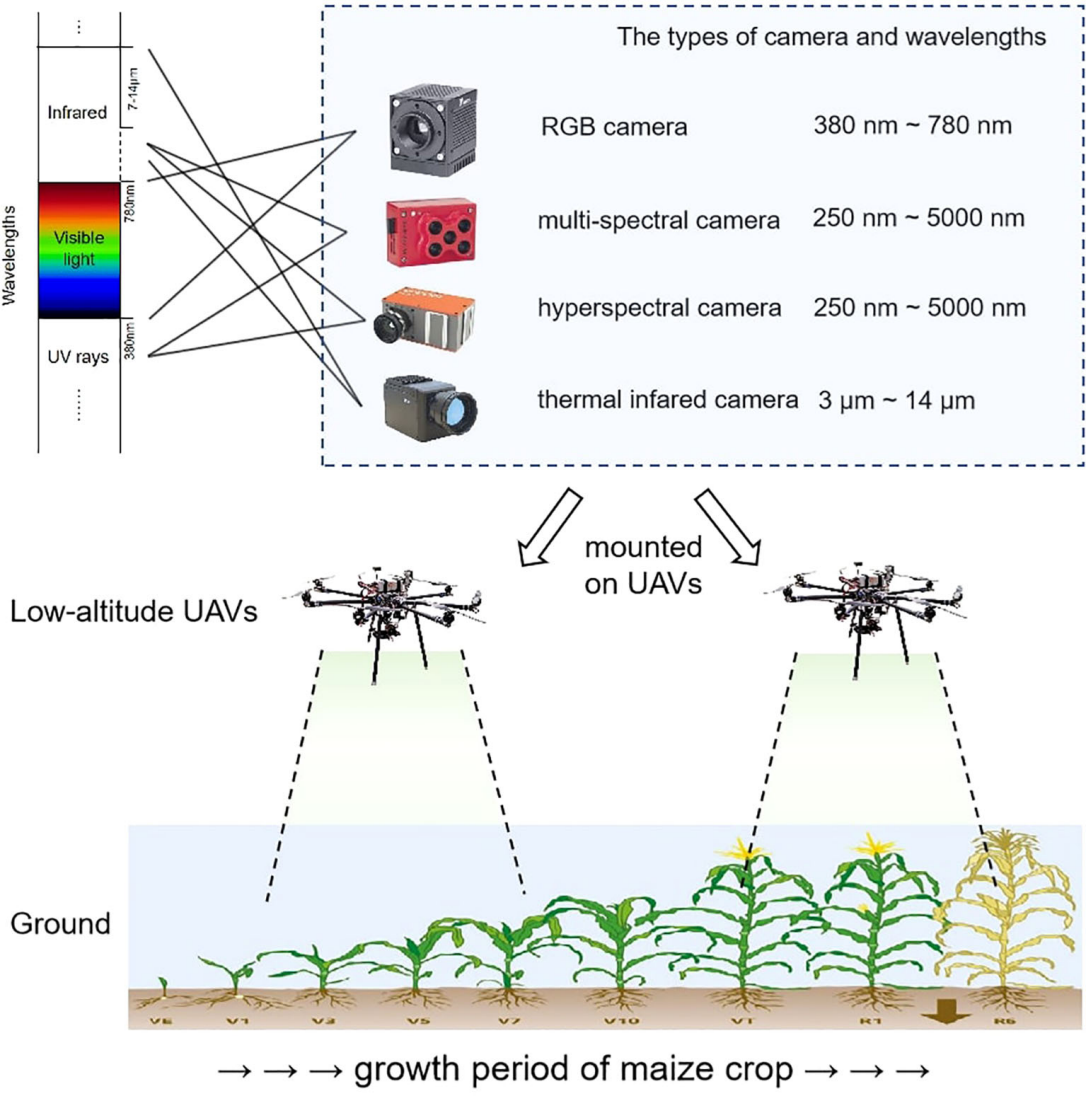
The UAV RS system mounted types of cameras.

Given that a variety of sensors are available for UAV systems, it is valuable to provide an overview of the cameras and sensors applicable to UAV systems and their characteristics. Colomina and Molina (2014) within their paper reviewed the specific parameters and applications of several RGB cameras, multi-spectral cameras, hyperspectral cameras, thermal cameras, and laser scanners adapted to UAVs, as can be seen in the literature. In pest and disease detection, especially the first four cameras are more applied in UAV RS, and these images are shown in **Figure 5**. Therefore, suitable solutions and deployment of UAV platforms can be found according to the research purpose and available budget.

#### 4.2.1 RGB cameras

RGB cameras measure specific intensities of the three-color channels of red (R), green (G), and blue (B) in the visible spectrum (400-780 nm), and the color of each pixel is expressed by the superposition of specific values of the three-color channels. RGB cameras have the advantages of being low-cost, easy to carry, and easy to operate, and has been widely used in plant phenotype research (Su, 2020). RGB images are usually processed and analyzed by ML or DL and have excellent application space in crop recognition, plant defect, and greenness monitoring due to their excellent performance (Dalsass et al., 2016; Darwin et al., 2021; van Iersel et al., 2018). Nevertheless, due to the limitation that the RGB cameras only contain three color channels in the visible light range and the poor spectral resolution, it is usually necessary to cooperate with other cameras to complete the identification and detection task.

#### 4.2.2 Multi-spectral cameras

Multi-spectral cameras are mainly used for the visible/near-infrared (VIS/NIR) region, which can obtain the advantages of both spatial information and spectral information of the detected targets. Rapid acquisition and analysis of crop growth information are achieved by analyzing changes in absorption, transmission, or reflection spectra in the visible (400-780 nm) and near-infrared (780-2500 nm) regions. The visible spectrum primarily conveys color-related information, whereas the near-infrared spectrum is derived from the molecular group’s vibrational absorption. When crops are afflicted by pests and diseases, a range of physiological and biochemical reactions take place, altering the composition and distribution of pigments, water, and other tissues in affected crops compared to their healthy counterparts. This leads to changes in the density and vibrational intensity of molecular bonds such as C-H, O-H, N-H, etc., which in turn cause variations in optical properties. It provides a theoretical basis for the wide application of multi-spectral cameras in crop yield, pest monitoring, and other fields (Ali et al., 2022). With the continuous development of cameras, the development of multi-spectral camera systems has derived many different types: Fourier transform base spectroscopy, wide and narrow band filters, and the like.

#### 4.2.3 Hyperspectral cameras

By capturing the spectral information of a target object at different wavelengths, the hyperspectral camera generates a data cube that contains both spatial and spectral information, allowing the user to analyze the target object from different angles and depths. Compared with the multi-spectral cameras, the imaging systems of the hyperspectral camera can collect more spectral bands, typically up to more than 100 spectral bands, and the hyperspectral cameras usually use one or more continuous wavelength ranges. This provides an incredibly detailed spectral signature for each pixel, enabling the identification of specific materials and chemicals. The use of hyperspectral RS can monitor the growth of crops in the field. By effectively integrating the obtained data, it can provide relevant information promptly, and applying this technology in predicting farmland disasters has a significant effect. Digital processing of images enables segmentation and classification of leaves in the corn fields of the Mexican fields making use of HIS color models (Carranza-Flores et al., 2020; Bravo-Reyna et al., 2020). Applying this mechanism to agricultural management can improve agricultural management and reduce the cost of agricultural management (Lu et al., 2020).

#### 4.2.4 Thermal infrared cameras

Infrared thermal imaging cameras detect infrared radiation emitted by objects, allowing for the measurement of their surface temperatures. They are sensitive to temperature differences and can detect subtle changes that are invisible to the naked eye. Infrared thermal imaging cameras are valuable tools for monitoring water stress in crops. Thermal infrared cameras operate by using the sensors they carry to capture infrared radiation in the range of 0.75 to 1000 µm emitted by the target object and feedback on the temperature of the target object as a digital thermal radiation image (Costa et al., 2013). When pests or pathogens infect crops, physiological states, for instance, transpiration, photosynthesis will change. Thermal infrared cameras can effectively monitor various characteristics, such as crop growth status and water stress, greatly contributing to IA (Calderón et al., 2013; Gago et al., 2015).

### 4.3 The technology road-map of RS

**Figure 6:**
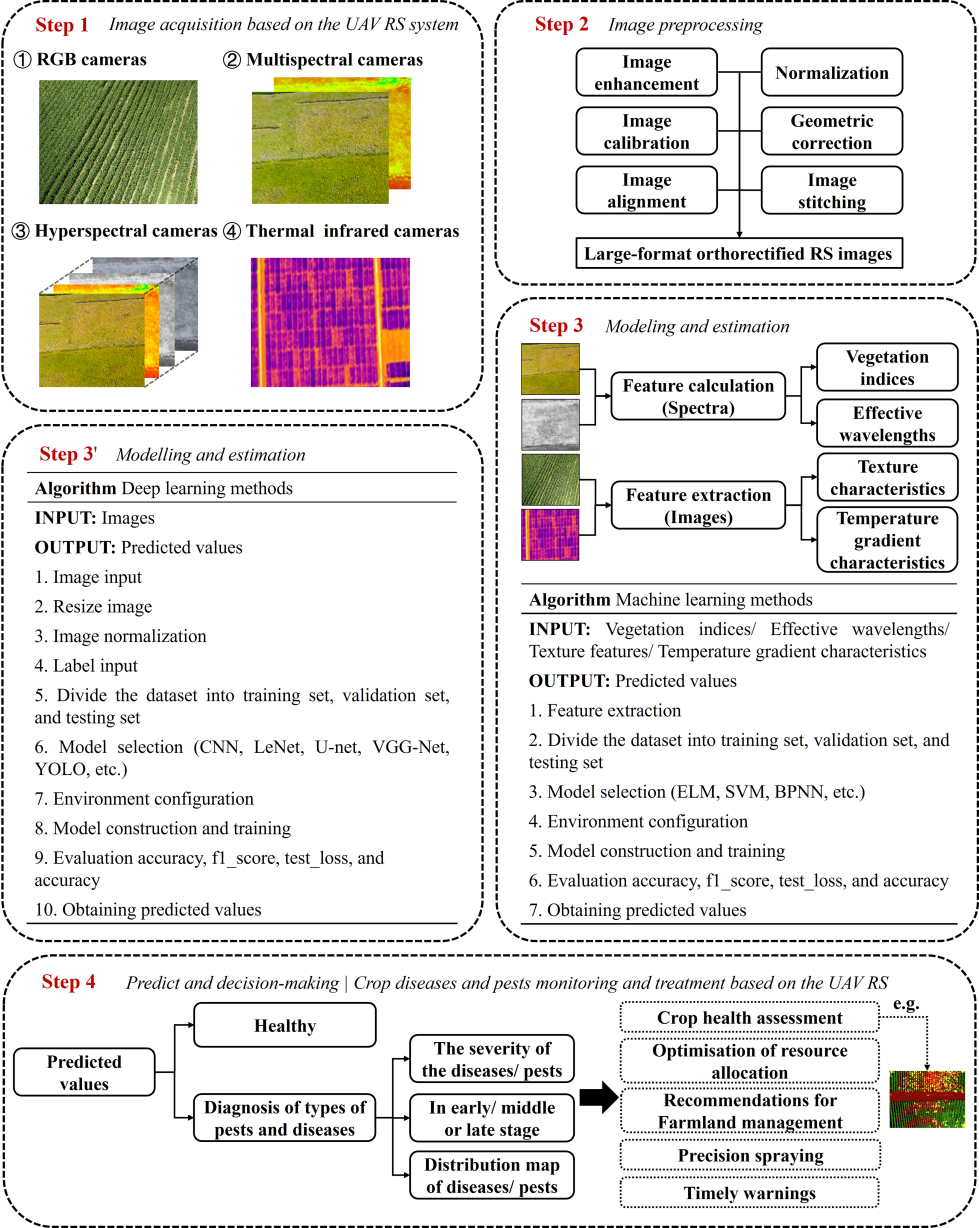
The main technology roadmap of crop diseases and pests monitoring.

From the acquisition of UAV RS images to the identification and location of crop diseases and pests, the whole process involves a series of complex processing processes. The main technology road-map of crop diseases and pests monitoring can be seen in **Figure 6**. The whole process includes image acquisition, image preprocessing, spectral feature extraction (including vegetation index, effective wavelengths), image feature extraction (texture characteristics, temperature gradient characteristics), modelling, prediction and evaluation using machine learning methods or deep learning methods, and finally using the predicted values to achieve monitoring and treatment of crop pests and diseases. Image preprocessing is necessary to improve image quality, remove noise interference in bad weather, simplify data and increase the accuracy and efficiency of subsequent image analysis and processing tasks. Because of the wide range of applications of spectral image sensors, this paper focuses mainly on spectral images. Traditional machine learning methods need to extract features before building a model and finally get the predicted values, while deep learning methods can build models directly from image inputs. Through qualitative analysis, it is possible to determine whether the crop is infected with a disease and what type of disease it is. Through quantitative analysis, it is possible to analyze whether the crop is in the early, middle or late stages of the diseases/pests infestation, the severity of the diseases/pests infestation, and most importantly, construction a map of the regional distribution of the diseases/pests infestation (Kerkech et al., 2020). In this way, we can realize crop diseases and pests monitoring and treatment based on the UAV RS. During the flight of the UAV, it will be affected by many factors, such as light, wind speed, and the state of the crop canopy. The collected RS data often have noise and interference information, which brings adverse effects to constructing the pest detection model (Alvarez-Vanhard et al., 2021).

Although many UAV RS systems are equipped with corresponding image processing software for noise processing, many noises are still in the processed RS data (Bunting, 2017). To eliminate the noises and interference information in RS data, improve the stability and signal-to-noise ratio of data, and obtain more helpful information for the detection tasks, savitizky-golay convolution smoothing (SG), variable normalization (SNV), multivariate scatter correction (MSC), wavelet transform (WT), and other predictive processing methods are often used to process the collected RS data (Yang et al., 2022). Nighttime images captured in hazy conditions often suffer from glow effects, poor lighting, reduced visibility, and considerable noise, leading to a significant decline in image quality. These compromised images can negatively impact subsequent computer vision processes. As a result, effective dehazing of nighttime images is crucial to enhance the clarity of such images and facilitate outdoor computer vision applications. To address this, a novel dehazing algorithm called the ‘multi-purpose oriented single nighttime image haze removal based on unified variational retinex model’ was introduced (Liu et al., 2023). This advanced unified variational retinex model processes a pre-treated nighttime hazy image by separating it into three components: reflectance, illumination, and noise. The method then individually enhances the reflectance and illumination components through dehazing and gradient domain enhancement techniques. Some crop diseases and pests detection categorize and methods are listed in **Supplementary Materials Table A1**.

## 5 Traditional machine learning in intelligent agriculture

Currently, the primary data analysis methods of UAV RS monitoring crop diseases and pests stress focus on the modeling analysis of traditional ML algorithms. The color and texture features of the diseased plants are obtained by using various sensors on the drone (gray level histogram, gray level co-occurrence matrix, wavelet transform, etc.). Temperature and humidity parameters, effective wavelengths, vegetation indices formed by fusing multiple spectral characteristics, and other parameters are used as model input variables. A qualitative or quantitative pest detection model is established by combining ML algorithms such as a support vector machine, a clustering algorithm, a random forest algorithm, a Bayesian algorithm, a least square method, etc. Ultimately, it can realize early discovery, species identification and classification of agricultural pests, and the grading of pest stress degree. It can provide efficient decision information for early prevention and control of crop diseases and pests.

Before using machine learning algorithms for simulation modeling, a feature extraction step is required. For different images, there are different processing methods. Spectral images are processed by feature calculation to get information such as vegetation indices and effective wavelengths. The processing method for RGB images as well as thermal imaging images is image feature extraction to obtain texture characteristics information along with temperature gradient characteristics information. Unlike deep learning methods, feature extraction is an indispensable step for traditional machine learning. Deep learning can directly input the image, but traditional machines need to input the information extracted from the features.

### 5.1 Features extraction methods

Feature extraction is one of the key steps in UAV remote sensing monitoring of pests and diseases, which involves extracting useful information related to pests and diseases from remotely sensed images. In general, feature extraction can include effective wavelength selection, vegetation index, and texture features. Effective wavelength selection algorithms refine fundamental indices from spectral data and exclude irrelevant spectral data collected by UAV RS systems, thereby improving model performance and simplifying calculations. The vegetation index is a parameter that can reflect the growth condition of vegetation, calculated by RS technology, especially multispectral and hyperspectral data. It can effectively synthesize the relevant spectral signals, enhance the vegetation information and reduce the interference of non-vegetation information. Changes in the vegetation index can reflect the health and vigor of crops, and is an important indicator for assessing the extent of the impact of pests and diseases. Texture features reflect the surface texture information of objects in UAV remote sensing images, and there are differences in leaf morphology, color and other aspects between damaged and healthy plants, which are manifested as different texture features on remote sensing images.

#### 5.1.1 Effective wavelengths selection

UAV RS systems with multi-spectral or hyperspectral cameras often gather extraneous spectral data, which can undermine the precision and consistency of pest detection models (Chen et al., 2002). Researchers have developed wavelength selection algorithms to distill essential indices from spectral data, enhancing model performance and simplifying computations (Thenkabail et al., 2000). These algorithms, chosen based on crop and pest spectral profiles, are tailored to specific detection contexts. Employing a blend of these algorithms or integrating additional ones could bolster model robustness, decrease errors, and boost prediction accuracy. This strategic application of wavelength algorithms advances detection capabilities, supporting targeted agricultural practices and crop safeguarding. Popular algorithms such as successive projections algorithm (SPA), genetic algorithm partial least squares (GAPLS), uninformative variable elimination algorithm (UVE), and competitive adaptive reweighted sampling (CARS) are in use (Li et al., 2016), with their effectiveness in detecting crop ailments and pests catalogued in research, exemplified in **Table 1**. for staples like wheat and soybeans. The t-test is a statistical method used to compare whether two sets of means are significantly different, while random forest (RF) is an integrated learning algorithm that makes predictions by constructing multiple decision trees. RF can be applied to select important wavelengths in spectroscopy by training the model on spectral data, extracting feature importance scores for each wavelength, and then choosing wavelengths with higher importance scores as they contribute more significantly to the model’s predictive performance.

**Table 1:** Application of the wavelength selection algorithms.

Sensors	Crop diseases/pests	Wavelength selection algorithms	Selected EWs (nm)	References
Hyperspectral cameras	Wheat powdery mildew	CARS	450, 560, 650, 730, 860	(Song et al., 2022)
Hyperspectral cameras	Aphid density of winter wheat	T-test	491, 617, 750, 1690	(Luo et al., 2013)
Hyperspectral cameras	Rice canopy infested with brown spot disease	GAPLS	822, 738, 793, 402, 570, 753	(Zhao et al., 2012)
Hyperspectral cameras	Strawberry anthracnose and gray mold	Random forest (RF)	822.93, 775.55, 783.26, 807.49, 890.28, 829.55,	(Jiang et al., 2021)
Hyperspectral cameras	The blight diseases of tomato leaves	SPA	442, 508, 573, 696, 715	(Xie et al., 2015)
Hyperspectral cameras	The gray mold disease of tomato leaves	T-test	655, 746, 756	(Xie C. et al., 2017)

#### 5.1.2 Vegetation indices extraction

In the research and practice of UAV RS detection, crop spectrum can reflect the growth status of crops. As a result, crop phenotype information can be obtained based on spectral reflectance, but sometimes the direct use of spectral reflectance cannot reflect the growth of plant canopy well. To solve this problem, many researchers try to optimize the crop-sensitive spectral reflectance to reconstruct the monitoring indices, enhance some characteristics and details of the vegetation, and highlight the difference between the detection targets. Twenty-five common in used extraction methods of vegetation indices from the RGB and multispectral camera, including 14 commonly used color vegetation indices and 11 multispectral narrow band vegetation indices are shown in Appendix **Supplementary Table A2**. The calculated vegetation indices were averaged over the sampled area. The color vegetation indices are visible band vegetation indices that highlight a particular color, such as the green color of plants, which is more intuitive to humans. Vegetation indices extracted from RGB cameras are sensitive to the greenness of plants, and they have been used to extract green vegetation and to calculate vegetation cover. Some improved vegetation indices such as ExG, Woebbecke’s index and some combined vegetation indices have also been used to explore the feasibility of predicting yields. First-order derivative spectroscopy is a technique used to remove background signal or noise and for resolving overlapping spectral feature establishment. It is also effective for enhancing the relationship between spectral data and target parameters. In practical scenarios, high-resolution images captured via UAVs can undergo processing by a color feature extraction algorithm to derive diverse color feature values. Subsequently, machine learning techniques are leveraged to develop models for detecting crop diseases and pests.

In addition, 68 commonly used vegetation indices were selected in **Supplementary Materials Table A3** regarding available literature. These vegetation indices can be broadly classified into several categories: characteristic, soil line, and atmospheric adaptive indices. Characteristic indices are mostly two or more band operations in the visible and near-infrared bands, such as simple two-band chlorophyll indices, normalized indices and so on. Soil line and atmospheric adaptation vegetation indices are designed to reduce the effects of environmental factors such as soil and atmosphere.

#### 5.1.3 Texture features extraction

As one of the important characteristic variables to characterize the growth of crops, texture features represent the spatial organization of pixel intensity changes in images and contain much essential information related to the occurrence of crop diseases and pests (Mardanisamani et al., 2019). **Supplementary Table A4** in **Supplementary materials** show 9 common texture characteristic parameter calculation methods. In practical applications, high-resolution images can be obtained by the UAVs and processed by a texture feature extraction algorithm to obtain values of different texture features. Then ML is applied to establish crop diseases and pests detection models. At present, the most commonly used texture features extraction method is to extract four texture features from RS images, namely, contrast CON (Contrast), correlation COR (Correlation), entropy EN (Entropy), and homogeneity HO (Homogeneity), according to gray level co-occurrence matrix (GLCM) (Wang et al., 2017). Combining texture features with their type feature variables can effectively improve the detection performance of quantitative detection models (Pang et al., 2021). In addition, the DL model also shows excellent performance in texture feature extraction. These depth models can automatically learn complex texture features, so they are widely used in the field of crop pest detection. When applying the depth model, a large amount of mark sample data is needed to train the model, which also becomes a big challenge in texture feature extraction by using the depth model.

### 5.2 Typical traditional machine learning frameworks

**Figure 7:**
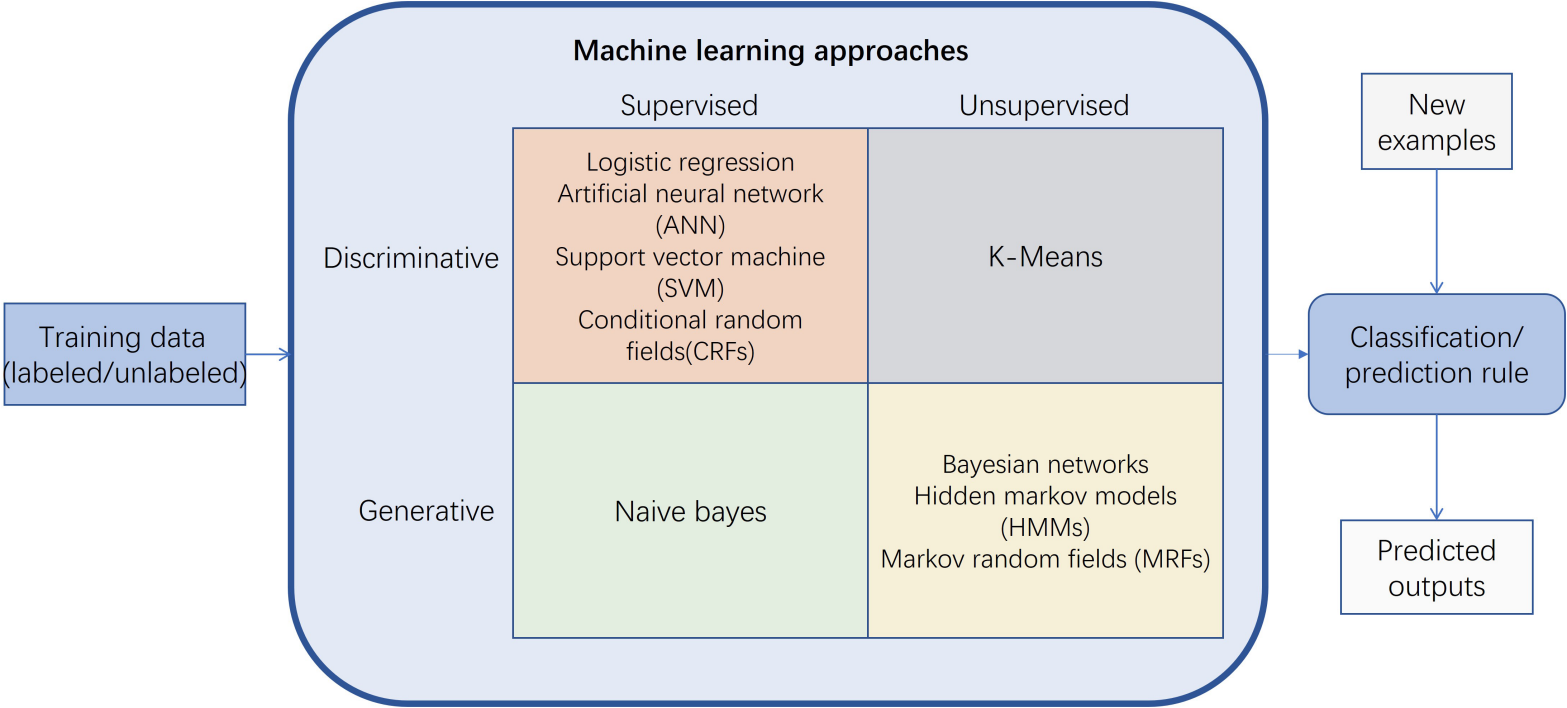
A typical machine learning approach system.

An overview of a typical ML approach system is illustrated in **Figure 7**. ML tasks start with input training data. Depending on the learning signal of the learning system, training data are typically sorted into two types: labeled and unlabeled. Hence, ML approaches are mainly classified into three categories: supervised learning, semi-supervised learning and unsupervised learning. Supervised learning involves the algorithm being trained on a data set complete with input variables and their corresponding output results, aiming to deduce a rule that consistently links the two. Sometimes, the inputs might be incomplete, or the desired outputs may only be provided as feedback within a changing environment, a scenario known as reinforcement learning. In such supervised scenarios, the model that has been honed is then applied to estimate the unknown outputs or ‘labels’ for new data. Conversely, unsupervised learning deals with data that lacks predefined labels, merging all data without a clear division into training or test subsets. Here, the algorithm’s goal is to sift through inputs and unearth underlying structures or patterns.

#### 5.2.1 Extreme learning machine

The ELM is highly effective in generalizing feed-forward neural networks. It utilizes a single hidden layer neural network that can randomly assign input weights and biases in the hidden layer, thereby addressing common issues such as over-fitting and local minima (Ding et al., 2014). The ELM stands out for its rapid learning pace, robust generalization capability, and ability to find unique, optimal solutions. As a result, it’s extensively employed in a variety of classification and regression tasks. In the research of Kouadio et al. (2018), ELM model has the ability to analyze soil fertility properties and to generate an accurate estimation of robusta coffee yield. The performance of 18 different ELM-based models with single and multiple combinations of the predictor variables based on the soil organic matter, available potassium, boron, sulfur, zinc, phosphorus, nitrogen, exchangeable calcium, magnesium, and pH, was evaluated. Khan et al. (2022) applied a multi-level deep entropy-ELM feature selection technique to recognize cucumber leaf diseases, and the best accuracy obtained by training on five different datasets was 98.4%.

#### 5.2.2 Support vector machine

The SVM identifies a hyperplane that maximizes the margin between the closest points of the training dataset. This process is executed through quadratic programming optimization using a radial basis kernel function, making it ideal for tackling small-sample, nonlinear, and high-dimensional datasets in both classification and regression contexts. SVM operates on the principle of structural risk minimization, constructing an optimal hyperplane for ideal classification by balancing training set error and complexity (Mahesh, 2020). SVM employs four primary kernel functions: the linear kernel, polynomial kernel, sigmoid function, and radial basis function kernel. These functions facilitate the transformation of low-dimensional space vectors into a high-dimensional space, aiding in the analysis of sample separability in this expanded space. Both regression and classification tasks in SVM share a common foundation. The choice of kernel function plays a crucial role in influencing the effectiveness of SVM in classification or regression. For classification tasks, SVM outputs a class value, whereas in regression scenarios, the output can be any real number. Kale and Shitole (2021) analyzed a SVM based method for crop disease detection. SVM algorithm is used to classify the extracted features. The data points are classified by finding a maximum spaced hyperplane in N dimensional space and the position of the hyperplane is optimized by means of support vectors in order to minimize the classification error.

#### 5.2.3 Back propagation neural network

The BPNN primarily utilizes an error back-propagation algorithm. This method involves adjusting the network’s connection weights after each training cycle based on the error back-propagation, continuing until the discrepancy between the actual and predicted output values is minimized (Hecht-Nielsen, 1992). For a given input sample set in BPNN, it’s necessary to define the number of hidden layers and nodes, select a learning algorithm and rate, choose a transfer function, and establish conditions for training termination. The neuron transfer functions in BPNN typically encompass a threshold function, a linear function, and a sigmoid function. The presence of irrelevant information in the data can impact the accuracy of the BPNN algorithm, and the inclusion of large sample data sets can slow down the BPNN’s modeling process. Consequently, it’s more efficient to build BPNN models using characteristic variables derived from raw data as inputs, which also enhances the computation speed.

## 6 Deep learning algorithms

In the modeling process of traditional ML algorithms, feature extraction mainly depends on a manually designed feature extractor, which requires solid professional knowledge and the ability to model parameter adjustment. Meanwhile, each algorithm has strong pertinence but poor generalization ability and robustness (Nazarenko et al., 2019). In contrast, deep learning relies on deep neural network (DNN) advantages, for example highly optimized algorithms and multiple unit layer architecture. DNN can extract features based on data-driven, automatically extract deep and dataset-specific feature representation according to the learning of a large number of samples, which is more efficient and accurate for the expression of the dataset. The extracted abstract features are more robust and have better generalization ability, which can realize end-to-end expression (Shrestha and Mahmood, 2019). With the explosive growth of UAV remote sensing image data, the importance of deep learning becomes more important than ML. Some state-of-the-art (SOTA) methods are the followings: convolutional neural network (CNN), VGG-Net (Simonyan and Zisserman, 2014), ResNet (He et al., 2016), ResNeXt (Xie S. et al., 2017), HRNet (Wang et al., 2020), RegNet (Radosavovic et al., 2020), LeNet, U-Net, etc. Therefore, DL algorithms have shown promising results and great potential in the early detection of crop diseases and pests, identification of pest species, and classification of disease severity (Kamilaris and Prenafeta-Boldú, 2018). Many researchers have conducted applied research on DL algorithms and achieved good results, as shown in the appendix **Supplementary Table A5**. The following describes a variety of DL frameworks commonly used in crop diseases and pests detection.

### 6.1 Typical deep learning frameworks

With the development of computer technology, advanced learning is represented by CNN (Abdel-Hamid et al., 2014). It extracts features layer by layer through convolution and pooling and has the characteristics of weight sharing and local connection at the same time, which reduces the number of training parameters and makes the model easier to optimize. After the proposal of CNNs, various new deep network structures have been proposed. In this article, the main focus is on three commonly used deep learning network structures found during previous hot-spot analyses.

#### 6.1.1 LeNet network

**Figure 8:**
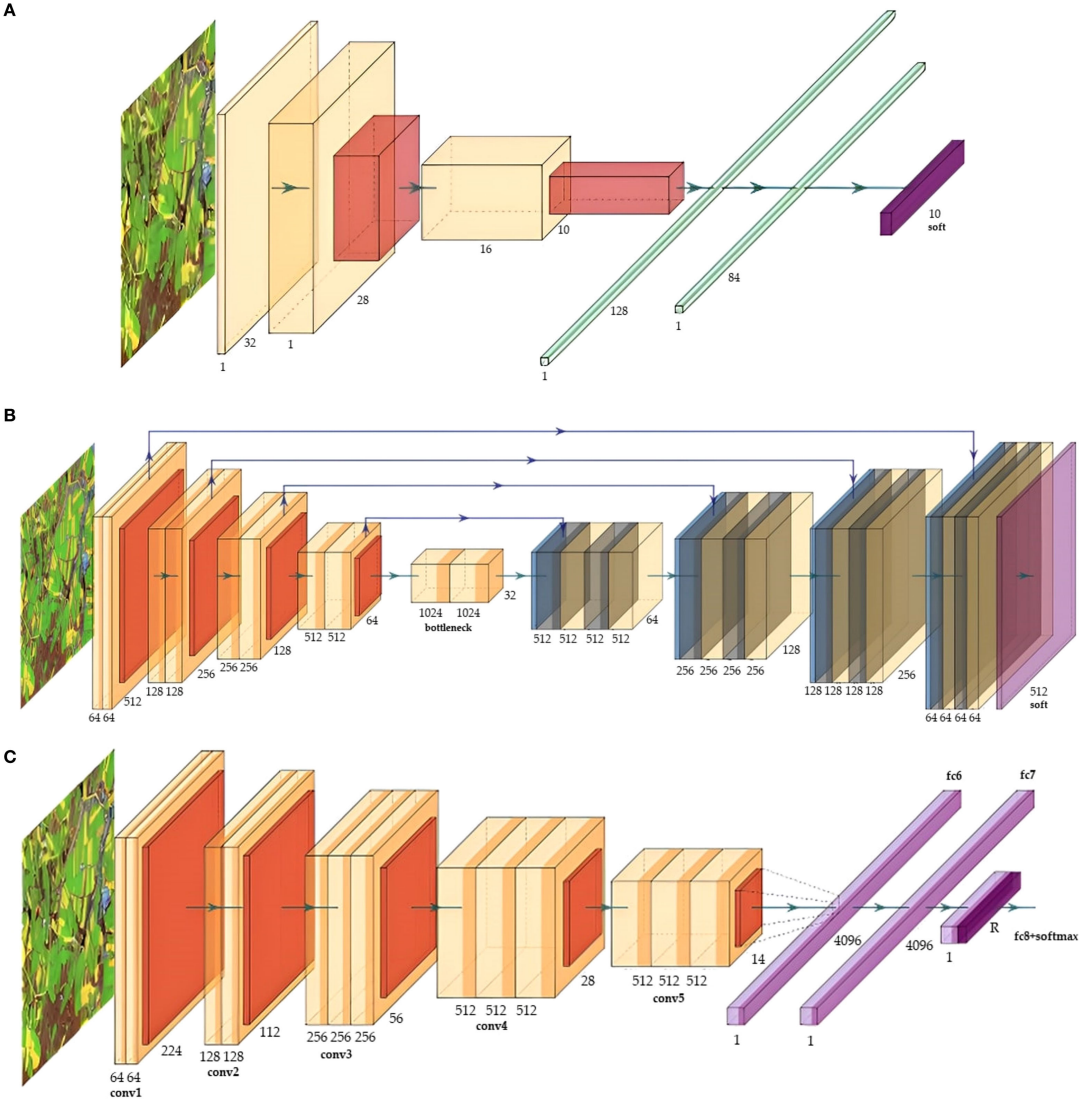
The deep learning models. **(A)** LeNet, **(B)** U-net, **(C)** VGG-Net.

LeNet is a DL framework proposed by LeCun et al. (1998). LeNet has seven layers of the network, including two convolutional layers, two pooling layers, and three fully connected layers. It uses softmax to process the output data. In addition to the input layer, each layer contains trainable parameters and has multiple feature maps. LeNet is the first successful application of CNN to solve the image classification problem, which is widely used in image recognition and text character recognition tasks. Though the network structure of LeNet is relatively simple, it has great adjustability in changing the size of the full connection layer and the size of the convolutional layer and can adapt to different image classification tasks. The feature map can extract each input feature through a convolutional filter, and its topology is illustrated in **Figure 8A**. In addition, modern convolutional neural networks are often improved and developed based on the LeNet framework. For example, classical convolutional neural networks such as AlexNet, VGG-Net, GoogLeNet, and ResNet have all proposed more effective network structures and training strategies on the basis of LeNet’s work and achieved amazing results in image classification and object recognition. Gayathri et al. (2020) used LeNet to classify and detect various diseases of tea, among which the accuracy rate of tea red spot disease was as high as 94%. Wallelign et al. (2018) obtained 12673 soybean images taken in the natural environment and used the LeNet framework to classify and detect many diseases, such as soybean septicemia and leaf blight, among which the highest accuracy rate reached 99.32%.

#### 6.1.2 U-net network

U-net was proposed by Ronneberger et al. (2015), and its original intention was to solve the problem of medical image segmentation. Later, due to its excellent performance, the U-net architecture became more popular in various fields. The U-net is composed of two main parts: the feature extraction layer and the up-sampling layer. Its name comes from its U-shaped architecture, and its topology is illustrated in **Figure 8B**. The feature extraction part consists of two 3x3 convolutional layers (ReLU activation function) plus a 2x2 max-pooling layer (maximum pooling layer) to form a down-sampled module right half, which is repeatedly composed of an up-sampled convolutional layer (deconvolution layer) plus feature concatenation concat plus two 3x3 convolutional layers (ReLU). To further improve the performance of U-net, many researchers have improved and extended the U-net network structure to adapt to more complex scenes. For example, the introduction of deeper network layers, attention mechanisms, multi-scale structure, cross-scale connection, and other technologies can improve the performance and generalization ability of the U-net network to some extent. Because of the excellent performance and flexibility of the U-net algorithm itself, as well as its abundant improvement and extension methods, its application prospect and research value in the field of computer vision are paid more and more attention. Bhujel et al. (2022) used the U-net model to perform image segmentation and quantitative analysis on strawberry gray mold, and the U-net model showed robust performance on a wide range of test images. Chen S. et al. (2021) employed the U-net architecture to subdivide rice bacterial leaf spot disease and evaluate the severity of leaf spot disease. Rao (2021) utilized the improved separable convolution of the U-net model to classify cassava mosaic disease and cassava bacterial wilt, and the accuracy reached 83.9%.

#### 6.1.3 VGG-Net network

VGG-Net is a model proposed by the visual geometry group of Oxford University. It is characterized by small convolution and deep network layers, which proves that increasing network depth can effectively improve performance. According to the size of the convolution kernel and the number of convolution layers, there are six configurations of VGG, namely A, A-LRN, B, C, D, and E, of which D and E are the most commonly used, namely VGG16 and VGG19. VGG16 has sixteen layers, including thirteen convolutional layers and three fully connected layers. The first time, after two convolutions with 64 convolution kernels, one pooling is used. The second time, after two convolutions with 128 convolution kernels, another pooling is used. Three 512 convolution kernels are repeated twice, and then pooling is repeated. Finally, three fully connected layers are used. The topology is shown in **Figure 8C**. Li et al. (2020) used the VGG16 and InceptionV3 to identify different degrees of ginkgo leaf disease. The accuracy of VGG16 in laboratory datasets was 98.5%, and the accuracy rate in the field dataset was 92.19%. Rangarajan et al. (2018) used the pre-trained DL architecture VGG16 net to classify tomato crop diseases. The classification accuracy of the VGG16 network using 13,262 images was 97.29%. Yan et al. (2020) employed an enhanced version of the VGG16 model to classify and identify apple leaf diseases. In this modified model, they replaced the fully connected layer with a global average polarization layer to decrease the number of parameters. Additionally, a batch normalization layer was incorporated to accelerate the convergence speed. The experiments conducted with this model demonstrated an overall accuracy of 99.01% in apple leaf disease classification.

### 6.2 Latest technology in processing crop diseases and pests imagery

Large language models (LLM) have been widely used in various fields with promising results. However, the IA has yet to fully integrate LLM into its practice due to the dominance of visual images. For this reason, it is worth exploring how LLM can be applied to IA on a large scale. It’s worth mentioning that the target detection network——the YOLO (You only look once) network was proposed as a fast-type method since it adopts a single neural network to run all components of the given task. YOLO is widely used for its small size and fast processing speed, and has been upgraded iteratively, the latest version is YOLOv10 (Wang et al., 2024). Qing et al. (2023) made a nice try to simultaneous application of YOLO and large models for pests and disease detection. A novel approach that combines YOLOPC and Gpt-4 was proposed. YOLOPC is a YOLO lightweight variant. Using the ability of YOLOPC, turning the affected leaves images into a natural language description of the disease, which is called image-to-text. After that, these descriptions of the crop diseases are then entered into a chatbot, which uses the reasoning capabilities of GPT-4 as well as the language generation capabilities to generate a disease diagnosis report. The experiments were tested and evaluated using datasets from different sources. Test results show that on the premise of entering text assistant, this network model’s induction and reasoning module demonstrates 90% reasoning accuracy in generating agricultural diagnostic reports. A very interesting and novel attempt to use existing models in combination with LLM. In the future, there will be more powerful LLMs such as GPT-5 or GPT-6, and this combination of ideas will still be very useful in the future.

**Figure 9:**
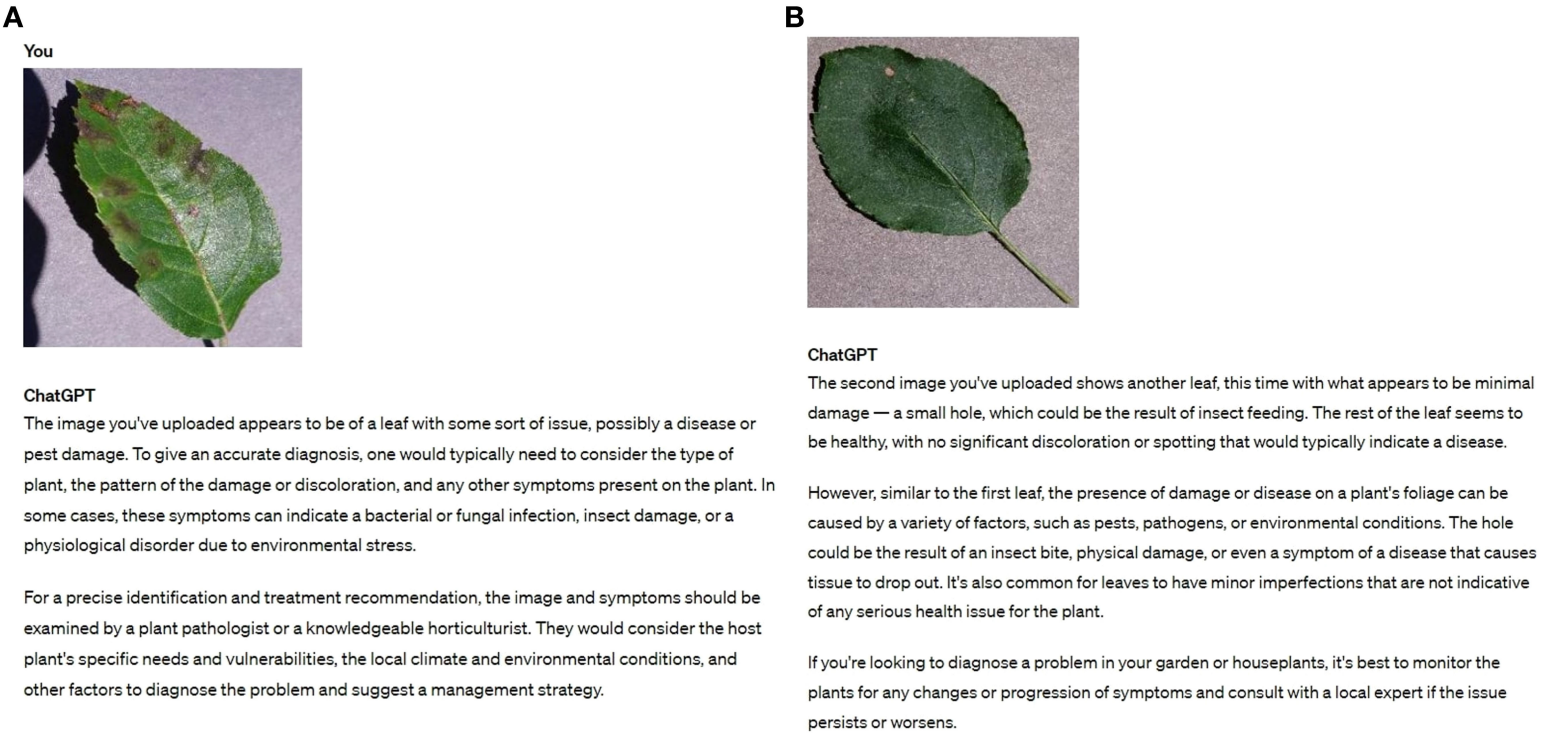
The GPT-4 results after entering an image of diseased leaves. **(A)** Severe diseases **(B)** Slight diseases.

The development of large vision models (LVM) is a direction that AI researchers want to follow to make further progress as they continue to refine LLM. Compared to LLM, visual information is typically 2-dimensional images, 3-dimensional stereo images, or 4-dimensional stereo video information. Therefore, the increase of 1-3 dimensions compared to the processing of linguistic information results in a higher difficulty level. However, the emergence of GPTs that are simultaneously LLM and LVM has changed our perception. It is capable of both processing image data and expressing the results obtained by processing the data in natural language to form an agricultural inspection report. As shown in **Figure 9**, we tried out the results of GPT-4’s reasoning with images of a diseased leaf. The image used was the apple leaves scab fungus disease image. We tested GPT-4 on images of one leaf that was severely diseased, and another leaf that was slightly diseased. The two paragraphs in the picture are ChatGPT’s responses to the two pictures I uploaded of diseased leaves. ChatGPT gives its judgement and advice. Notably, the image of the slightly diseased leaf posed challenges for recognition. The outcomes revealed the capability of GPT-4 to successfully detect visibly diseased leaves. However, it fell short in effectively identifying subtle signs of disease in the slightly diseased leaves, providing less satisfactory results in such cases.

### 6.3 Deep learning in UAV RS - some discussions in detail areas

It is undeniable that DL-based methods are powerful and efficient tools for processing the large amounts of data generated by RS systems. In this section, there are some brief discussions of some detail areas.

#### 6.3.1 Image resolution problem

The input of DL architecture is low-resolution images, and the processing method for high-resolution images is to scale them to the size required for processing. Hence, it is a challenge that maintain the high resolution of images in the process. In this regard, the latest HRNet (Kannojia and Jaiswal, 2018) attempts to maintain high-resolution CNN architecture in deeper layers.

#### 6.3.2 Real-time processing

In the era of IA, crop protection applications can benefit from DL. However, these DL algorithms are highly computer-intensive. Usually, a data center or graphics processing unit (GPU) with strong computing power is required for post-processing. Therefore, the bottleneck of the development of DL in the field of IA is still real-time processing. There are two ways to solve this problem: developing better and faster algorithms and enhancing better GPUs. In terms of algorithms, MobileNets (Howard et al., 2017) with layers with fewer parameters can still maintain prediction performance. In terms of hardware, some platforms such as NVIDIA’s Jetson has been developed to better run DL (Mittal, 2019).

#### 6.3.3 Dimensional reduction

Unlike RGB images, the latest hyperspectral images captured by drones are composed of dozens to hundreds of spectral bands, which can help classify and regress the characteristics of leaves. However, high dimensionality brings two problems: First, the high correlated bands. Second, the increasing learning cost. Therefore, hyperspectral data may hinder the accuracy of DL-based methods, which is an important issue to consider in RS practice. At present, the classic method to solve high dimensions is to apply PCA, but today when the amount of data has increased significantly, this method may need to be updated. There, Licciardi et al. (2011) suggested a singular, integrated method within a network engineering framework. This method focuses on identifying the most pertinent frequency band combinations from hyperspectral sensors, directly correlating to the labeled data presented in the input layer at the network’s initial phase.

#### 6.3.4 Transfer learning

Transfer learning is the ability to learn to draw inferences from one example and learn new knowledge by using existing knowledge. Its core is to find the similarity between existing knowledge and new knowledge. How to reasonably find the similarities between them and then use this bridge to help learn new knowledge is the core issue of transfer learning. In practical applications of this model, there are frequently spectral shifts observed between the training and testing images. These shifts typically arise from variations in image acquisition, geographical locations, and atmospheric conditions (Tuia et al., 2016). Therefore, how to reduce the impact of the difference between actual indicators on the DL algorithm is also a direction worth studying.

## 7 Practical application of UAV RS

### 7.1 Application of the UAVs assembled the RGB cameras

The RGB cameras equipped with the UAVs are often used to obtain morphological indicators of crops, for instance, lodging area, leaf color, plant height, canopy coverage, and panicle number. Morphological indices, as important phenotype parameters to characterize crop growth, are essential in monitoring crop diseases. The RGB images are processed by RS image classification, image feature extraction, and hybrid model recognition, which can realize the rapid acquisition and accurate diagnosis of crop disease information.

Liu et al. (2018) constructed a wheat disease detection model based on a random coefficient regression model based on RGB RS images of UAV and realized an accurate evaluation of wheat powdery mildew disease severity. Oh et al. (2021) extracted crop canopy coverage, canopy volume, and vegetation indexes based on UAV RGB RS images and combined them with various ML algorithms to construct various detection models of corn tar spot disease, realizing accurate detection of corn tar spot disease. Calou et al. (2020) monitored banana yellow leaf disease with an RGB camera mounted on UAV and constructed the banana yellow leaf disease detection model by using ML algorithms including maximum likelihood, minimum distance, SVM, and artificial neural network. Among the above models, the overall accuracy of the SVM model achieved 99.28%. RGB cameras have the advantages of low prices and easy operation. Nevertheless, it is limited by the limited spectral band in RGB images, which cannot reflect more physiological information about crops. Compared with RGB cameras, UAV multi-spectral imaging systems can obtain more diverse spectral characteristics and perform better in biochemical trait estimation due to the contribution of near-infrared spectral information.

### 7.2 Application of the UAVs mounted the multi-spectral cameras

The multispectral imaging sensors carried by the UAVs obtain the map information of the crop, which can more comprehensively present the spectral characteristics of the crop and realize the *in-situ*, rapid, and efficient monitoring and accurate acquisition of the crop disease information. High spectral-resolution sensors have significant advantages and wide application potential in quantitative RS of IA.

Marin et al. (2021) extracted various vegetation indices including the green ratio vegetation index (GRVI), green normalized difference vegetation index (GNDVI), and normalized difference vegetation index (NDVI) based on UAV multispectral images. They constructed various coffee leaf rust detection models using four decision tree models (logistic model tree (LMT), reduced-error pruning tree (REPTree), regression tree (RT), and RF), among which the LMT detection model achieved 91.5% accuracy. Lan et al. (2020) used the UAV RS platform to collect multispectral RS images of large citrus orchards, explored the best vegetation index combination for citrus Huanglongbing detection, and constructed various citrus Huanglongbing detection models in combination with various ML algorithms (SVM, k-nearest neighbors (KNN), naive Bayes, logistic regression, ensemble learning). Xiao et al. (2022) constructed various apple fire blight detection models based on UAV multispectral RS images and various ML algorithms (decision tree, RF, and SVM), among which the overall accuracy of the random forest model reached 94.0%. Zheng et al. (2018) designed and proposed a real-time wheat disease detection algorithm called efficient double-flow UNet (DF-UNet) based on UAV multispectral images and a DL algorithm for the diagnosis of wheat yellow rust severity. Compared with the jagged spectral map provided by the multispectral cameras, the hyperspectral cameras can provide a smooth spectrum and higher spectral resolution, which makes up for the defect that the multispectral cameras cannot depict narrow spectral features. In addition, the hyperspectral cameras’ imaging speed is faster, making the data acquisition cycle shorter and more efficient.

### 7.3 Application of the UAVs mounted the hyperspectral cameras

Hyperspectral imaging mainly uses narrow electromagnetic bands to obtain the spectral information of ground objects. Since it came out, it has been widely used in crop diseases and pests. Zhang et al. (2003) identified and detected tomato leaf miners based on hyperspectral fusion, and the recognition rate and overall recognition rate of the GA-BPNN model for samples at all levels reached 93.33%. Based on the hyperspectral RS of UAV, Feng S. et al. (2021) constructed the classification and detection model of leaf blasts and explored the classification method of rice leaf blasts, among which the classification prediction accuracy reached 98.58%. Ma et al. (2021) conducted hyperspectral RS on a chestnut planting area using a UHD185 hyperspectral camera mounted on a UAV (DJI Dajiang M600). They analyzed the spectral characteristics of leaves that were locally infected, unevenly infected, and recovering from infection. Then they determined the relationship between the damage degree of red spider diseases and pests and the changes in 6 spectral characteristics, namely the green peak, red valley, low position, red edge, high position, and high shoulder. Abdulridha et al. (2020) used unmanned aerial vehicles (Matrice 600 Pro, Hexacopter, DJI Inc.) with a hyperspectral camera to detect powdery mildew at different stages in pumpkins. In field conditions, the RBF neural network was used to classify the early and late stages of disease development as 89% and 96%, respectively.

### 7.4 Application of the UAVs mounted the thermal infrared cameras

The upper limit of the spectral range that the above three sensors can collect is 5000 nm, while the thermal infrared camera can collect spectral information between 3000 nm and 14000 nm. The thermal infrared cameras can collect crop temperature information, photosynthesis, transpiration, and other crop physiological conditions that cannot be obtained by the above cameras, providing another crop pests monitoring method.

The real-time monitoring of crop temperature using thermal infrared cameras mounted on UAVs is crucial for the detection of early pests and diseases with non-visual symptoms, and thermal images from UAVs have greater temporal and geographic resolution than images received from satellites, making them a valuable source of information for agronomic applications (Ribeiro-Gomes et al., 2017). Feng et al. (2022) used a six-rotor UAV (Matrice600PRO DJI) as an RS platform equipped with thermal capture real-time monitoring of wheat canopy temperature information by thermal cameras to classify and detect wheat powdery mildew. Chen A. et al. (2021) used UAV to obtain thermal and visible-light aerial images. Thermal imaging can effectively detect late blight and white mold in field crops.

### 7.5 Application of multi-source data fusion

Limited by the different band ranges, interference conditions, imaging mode, and mechanism obtained by each sensor, any single data source has limitations and cannot fully reflect the spatial-temporal characteristics of crops. The multi-source RS carried by UAVs acquires RGB images, full-band spectrum or multi-band spectrum information, three-dimensional structure information, and temperature of crops by carrying various crop information monitoring equipment. It realizes the joint application of multi-source monitoring information through data fusion and forms a complete working system of UAV RS monitoring crop diseases and pests.

Because of the complexity and diversity of the actual farmland layout and the seriousness of spectral mixing, it is imperative to improve the spatial resolution of UAV RS. Multi-source data fusion can effectively improve the ability to describe the details of crop spatial distribution, improve the spatial resolution and clarity of the image, and reduce the impact of mixed pixels to a certain extent. Image fusion is the most common multi-source RS data fusion method. It aims at improving the spatial resolution. The synthetic image with new spatial and spectral characteristics is generated by processing and calculating the multi-source RS data according to specific rules. The hue-luminance-saturation (HLS) transform method based on color correlation and the PCA, wavelet transform methods based on statistical methods can effectively improve the spatial resolution of UAV RS. In addition, grain characteristics, spectral characteristics, and biochemical characteristics produced by crops at different stages of suffering from different diseases and pests are significantly different. Therefore, long-term RS data collection and multi-temporal data fusion can make up for the deficiency in the timing of crop monitoring and greatly improve the accuracy of classification monitoring of crop diseases and pests.

For wheat powdery mildew, Feng Z. et al. (2021) fused the hyperspectral features collected by the spectral radiometer, thermal infrared image data obtained by a thermal infrared camera, and texture features obtained by an RGB camera, which greatly improved the intensive reading of RS monitoring of wheat powdery mildew. DadrasJavan et al. (2019) constructed a citrus Huanglongbing detection model based on the low-altitude RS platform of RGB and multispectral cameras carried by UAV, combined with the SVM algorithm, and achieved 81.75% overall classification effect. In their study, Lei et al. (2021) utilized RGB and multispectral RS images obtained by UAV to extract normalized difference vegetation indices, optimized soil adjusted vegetation index (OSAVI), leaf chlorophyll index (LCI), GNDVI, and normalized difference red edge (NDRE) index. They constructed different detection models for the injury levels of areca yellow leaf disease depended on five ML algorithms (BPNN, decision tree, naive Bayes, SVM, KNN). The results demonstrated that the classification accuracies of the test sets of the BPNN algorithm and SVM algorithm were satisfactory, at 86.57% and 86.30%, respectively. Francesconi et al. (2021) used an unmanned DJI Matrice 600 multi-rotor aircraft equipped with a Zenmuse X5 RGB camera and a Zenmuse XT thermal infrared camera to collect RGB images and thermal infrared images for data fusion, which improved the accuracy and efficiency of classification and detection of wheat fusarium cephalosporins. Based on the fusion of the hyperspectral image and the multispectral image source, Cock et al. (2016) established a monitoring model for corn borer damage, effectively classified areas with different disease severity levels, and facilitated exemplary management of corn planting areas.

## 8 Conclusion and prospects

### 8.1 Deficiencies in the existing research and challenges to be solved

In recent years, with the continuous innovation of UAV RS, outstanding platforms have been built for research in crop monitoring. The UAVs have made significant progress in detecting and classifying crop diseases and pests and greatly promoted the development of IA (Hunt and Daughtry, 2018). However, there are still some deficiencies in the existing research, and there are still many problems and challenges to be solved.

#### 8.1.1 Multi-source data fusion

There are a bunch of sources of monitoring data on crops, such as images or digital data obtained by hand-held spectral camera detectors, image data captured by cameras mounted on unmanned vehicles, and images captured by various types of cameras on UAVs. It’s worth exploring how to fusion these types of data because it will help us form a better overall system.

On the other aspect, due to the diversity of crop diseases and pests damages, different diseases and pests may appear simultaneously in similar spectral characteristics in the same crop. The same disease and pests may also appear in different spectral characteristics in the same crop at different times: the so-called ‘same spectrum foreign matter’ and ‘same substance different spectrum’ phenomenon. At the same time, a single sensor is limited by its spectral band, resolution, and other factors (Gao et al., 2020). The information on crop diseases and pests obtained by it is relatively limited, so it is difficult to solve the above problems. In contrast, multi-source data fusion can fully use different sensors’ characteristics and advantages. It can coordinate multiple sensors to jointly monitor crops’ growth, analyze crops’ growth from multiple levels, and provide a new solution for solving the errors and interference caused by the phenomenon of the same spectrum of foreign matter and the same thing different spectrum.

#### 8.1.2 Combination of multi-scale RS methods

The RS of UAVs is limited by the load capacity and endurance of the UAV itself, so it is challenging to monitor farmland in real time in large areas and long time. Therefore, how to improve the UAV RS to achieve multi-temporal and multi-space crop diseases and pests monitoring has become a momentous research topic. To solve this problem, multi-scale RS monitoring of crop diseases and pests, which combines space RS, aerial RS, ground RS, and UAV RS, is a more prominent solution.

RS monitoring by using sensors carried on satellites can obtain three characteristics spatial resolution, time resolution, and spectral resolution at the same time. It combines the temporal dynamic change of crop diseases and pests process, the spatial range change of pests and diseases occurrence area with spectral characteristics. It has the technical advantages of all-weather, multi-mode, and multi-polarization (Neupane and Baysal-Gurel, 2021). Additionally, aerial RS and ground RS data have the advantages of high spectral resolution and convenient and diverse acquisition methods, which entirely make up for the shortcomings and defects of UAV RS. By utilizing the plurality of RS technical means, the trinity of ground-space satellite and the point-surface combination is realized to monitor and classify crop diseases and pests together. Such a combination allows for multilevel monitoring of crop leaves and canopies. Thus, the method has extremely high practical value.

#### 8.1.3 Improvement of algorithms

In processing and analyzing the acquired UAV RS data using the traditional ML algorithm, tedious feature extraction operations need to be carried out so that real-time processing of the UAV RS data is difficult to realize. Meanwhile, the period of building a model is prolonged. However, using DL for modeling analysis requires much data to train the model. When the training data is insufficient, or the data sample type is unbalanced, problems such as under-fitting or over-fitting are prone to occur. In addition, the generalization ability of the DL model is weak. It is difficult to directly apply the model for specific crop pests to the pest recognition of other crops. Because of the above problems, the traditional ML algorithm is combined with the DL algorithm, and by utilizing the characteristics of the DL algorithm to automatically learn and extract the deep feature information of the data, the key feature information about the crop diseases and pests in the RS data is efficiently obtained, and the key feature information is taken as the input variable of the traditional ML algorithm so that a disease and pest detection model with stronger universality and stability is constructed.

### 8.2 Future of deep learning in IA

DL is still considered a ‘black box’ solution to most problems (LeCun et al., 2015), although new research is minimizing this concept to a considerable extent. Due to the complexity of layer number issues and parameter issues in deep learning, it is difficult for us to clearly understand why solutions that are considered good from the results are good. Regardless, in the field of RS, it has provided important discoveries in most of its implementations. In this paper, our aim is that this review of the literature will act as a comprehensive overview, encapsulating the various applications of UAVs in the realm of DL networks.

Therefore, from the above analysis, we can draw some conclusions:

1. It is necessary for drones to obtain additional labeled publicly available datasets for training and benchmarking networks.
2. Methods such as R-CNN and XGBoost are slightly insufficient in detection accuracy. However, emerging algorithms such as U-Net and PSNet have achieved better accuracy, which means there is still a lot of room for continuous improvement in algorithms.
3. DL can provide fast inference solutions with the assistance of GPU processing. However, further investigation is still needed regarding the real-time processing of UAVs using embedded systems.
4. In the context of UAV RS, whether some promising topics. For example, combining multi-head attention-based mechanisms and multi-task learning to form a new networks. Whether general large model technology can be applied to pest and disease detection in IA under UAV RS is also worth studying.

### 8.3 Something even better than DL in the near future

Since the AI application AlphaGo (Silver et al., 2017) defeated the world champ Jie Ke in May 2017, AI has become the talk of the town. Furthermore, the release of ChatGPT (Brown et al., 2020) and GPT-4 (Achiam et al., 2023) exacerbated this process. Maybe AGI could be useful in IA. General AI refers to the capability of machines to mimic human cognition and execute a wide range of tasks through transfer learning and other methods. GPT-4 exemplifies this as a multimodal system, combining language and visual recognition, allowing it to understand and process images. It is feasible to use LLM e.g. GPT4 for image recognition, text organization, and inference to generate crop diseases and pests diagnostic reports with suggested solutions (Lu et al., 2023).

The image processing process of traditional AI models is to analyze, simply reason, and draw conclusions. However, AGI models can recognize and analyze the image directly through ‘prompts’ and begin to reason, search the knowledge base, draw organized conclusions, and give readable scientific advice. This is the question-answering and dialogue system. Not everyone knows how to use AI models. However, the natural language conversation capability that AGI has allows these individuals to utilize AGI models using a spoken question-and-answer format. Assuming that AGI can be integrated into mobile, it would be a real convenience for a large number of farmers. In addition to this, there are many applications of knowledge graphs to agricultural AGI. For example, a search engine designed for farmers. Agricultural search engines enhance the connection between farmers and experts, providing easier access to necessary information. Additionally, agricultural recommender systems play a pivotal role. These systems aid in distributing reliable information, assisting farmers in making well-informed choices to boost production. This includes recommendations on the best agricultural inputs like seeds, fertilizers, and pesticides to use. By utilizing auxiliary information from the knowledge graph, user preferences can be captured more accurately, enabling more precise recommendations.

AI service integration holds great potential for assistance, documentation, education, interpretation forecasts or data-driven predictions. LLM depict a fundamental step to reduce the gap between AI-driven data analysis and common users. For agriculture, this implies that LLM will improve farmer consultation by providing all necessary information e.g. crop cultivation, breeding, machines, or phytopathology to an advisor or the farmer directly (Kuska et al., 2024).

**Figure 10:**
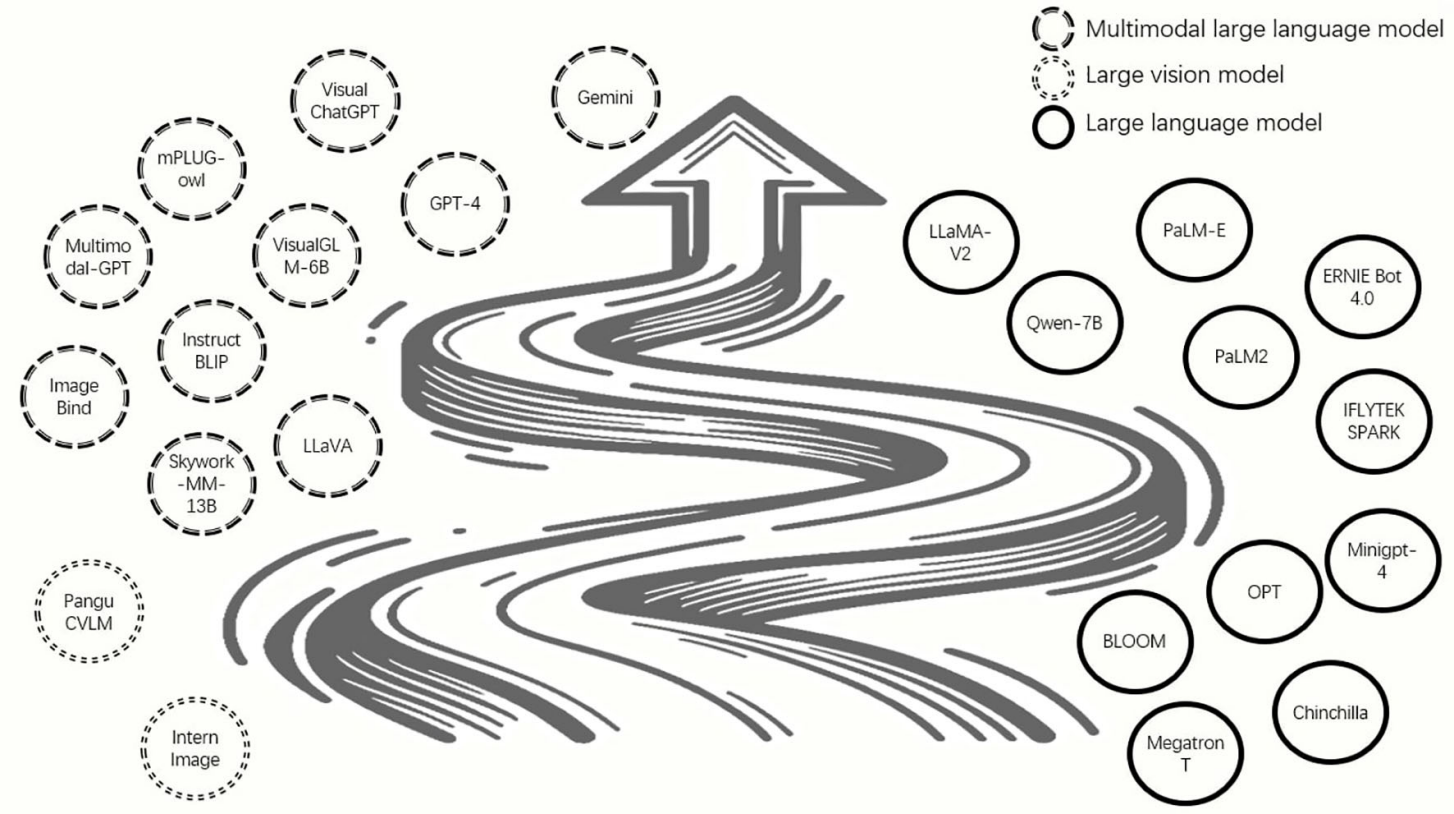
LLMs/LVMs/MLLMs that could be used on IA in the future.

As shown in **Figure 10**, we summarized the development process of the big models and selected some models released in 2022 and 2023. The overall trend in large models is from LLM to LVM to multi-modal large language model (MLLM). On one hand, the use of LLM can be combined with other computer vision techniques. On the other hand, the use of LVM could process UAV RS imagery directly. Further on, the MLLM is capable of inputting text, sound, image, and video, and also outputs the multimodal items. The aforementioned LLM, LVM, and MLLM, are all belong to AGI. Considering the generalization of AGI, all these models mentioned in the Figure can be applied to IA in the future. However, for the time being, LLM still has a number of limitations. The outputs generated by LLM sometimes do not provide valid answers due to insufficient information. Often the source of data used to generate the output is not clearly known, affecting the reliability of the output. The performance of multilingual extensions is low, especially in agricultural applications, which are more complex when different languages, dialects and technical terms are involved. Recommendations and conclusions that are valid in one region may not be applicable when translated to another region with different geographical, legal and agricultural conditions.

## Abbreviation

IA: intelligent agriculture
UAV: unmanned aerial vehicle
RS: remote sensing
AI: artificial intelligence
DL: deep learning
ML: machine learning
LLM: large language model
LVM: large vision model
FAO: food and agriculture organization
ELISA: enzyme-linked immunosorbent assay
IF: immunofluorescence
PCR: polymerase chain reaction
FISH: fluorescence *in situ* hybridization
AGI: artificial general intelligence
WOS: web of science
PCA: principal component analysis
VIS: visible light
NIR: near-infrared
SG: savitizky-golay convolution smoothing
SNV: standard normal variate
MSC: multivariate scatter correction
WT: wavelet transform
CON: contrast
COR: correlation
EN: entropy
HO: homogeneity
GLCM: gray level co-occurrence matrix
SPA: successive projections algorithm
GAPLS: genetic algorithm partial least squares
UVE: uninformative variable elimination algorithm
CARS: competitive adaptive reweighted sampling
RF: random forest
ELM: extreme learning machine
SVM: support vector machine
BPNN: back propagation neural network
DNN: deep neural network
CNN: convolutional neural network
SOM: self-organizing map
LLM: large language model
YOLO: you-only-look-once
LVM: large vision model
GPU: graphics processing unit
GRVI: green ratio vegetation index
GNDVI: green normalized difference vegetation index
NDVI: normalized difference vegetation index
LMT: logistic model tree
REPTree: reduced-error pruning tree
RT: regression tree
KNN: k-nearest neighbors
DF-UNet: double-flow UNet
HLS: hue-luminance-saturation
OSAVI: optimized soil adjusted vegetation index
LCI: leaf chlorophyll index
NDRE: normalized difference red edge
MLLM: multi-modal large language model.
