# Supplementary material for: Intelligent agriculture: deep learning in UAV-based remote sensing imagery for crop diseases and pests detection
Source: Front Plant Sci. 2024 Oct 24;15:1435016. doi: 10.3389/fpls.2024.1435016 (PMC11540708; doi:10.3389/fpls.2024.1435016)
Supplement: Supplementary file 1 [file Table1.docx]

**Supplementary materials**

**Table A1.** Types of crop diseases and pests detection methods.

|  | **Categorize** | **Methods** | **Reference** |
| --- | --- | --- | --- |
|  |  |  |  |
| Crop diseases and  pests damages | Coffee | ELM, RF, MLR | (Kouadio et al., 2018) |
|  | Maize | RF, ANN | (Pineda et al., 2018) |
|  | Recognition of 10 tomato plant diseases and pests | CNN | (Fuentes et al., 2018) |
|  | Avocando iron deficiencies | DT, MLP | (Abdulridha et al., 2018) |
|  | Identification of six plant leaft diseases | RBFN | (Chouhan et al., 2018) |
|  | Identification of tea red scab, tea leaf blight and tea red leaf spot diseases | SVM, DT, RF, CNN | (Dhingra et al., 2019) |

**Table A2.** Vegetation indices derived from RGB and multispectral images.

| **Vegetation indices** | **Formula** | **References** |
| --- | --- | --- |
| **RGB-** **Colour vegetation index** | | |
| visible-band difference vegetation index, VDVI | $VDVI=(2G-R-B)/(2G+R+B)$ | (Hunt et al., 2013) |
| visible atmospherically resistant index, VARI | $VARI=(G-R)/(G+R-B)$ | (Gitelson et al., 2002a) |
| normalized green-red difference index, NGRDI | $NGRDI=(G-R)/(G+R)$ | (Motohka et al., 2010; Gitelson et al., 2002a) |
| normalized green-blue difference index, NGBDI | $NGBDI=(G-B)/(G+B)$ | (Du & Noguchi, 2017) |
| red-green ratio index, RGRI | $RGRI=R/G$ | (Verrelst et al., 2008) |
| green-red ratio index, GRRI | $GRRI=G/R$ | (Du & Noguchi, 2017) |
| modified green red vegetation index, MGRVI | $MGRVI=(G^{2}-R^{2})/(G^{2}+R^{2})$ | (Bendig et al., 2015) |
| excess green index, ExG | $ExG=2G-R-B$ | (Woebbecke et al., 1995) |
| color index of vegetation, CIVE | $CIVE=0.441R-0.881G+0.385B+18.78745$ | (Torres-Sánchez et al., 2014) |
| vegetativen, VEG | $VEG=G/(R^{a}\times B^{(1-a)})，a=0.667$ | (Hague et al., 2006) |
| excess green minus excess red, ExGR | $ExGR=ExG-ExR=ExG-1.4R-G$ | (Meyer & Neto, 2008) |
| woebbecke index, WI | $WI=(G-B)/(R-G)$ | (Woebbecke et al., 1995) |
| combination, COM | $COM=0.25ExG+0.3ExGR+0.33CIVE+0.12VEG$ | (Guijarro et al., 2011) |
| combination 2, COM2 | $COM2=$0.36ExG+0.47CIVE+0.17VEG | (Guerrero et al., 2012) |

**Table A2. (continued).**

| **Vegetation indices** | **Formula** | **References** |
| --- | --- | --- |
| **Multispectral narrow-band vegetation index** | | |
| simple ratio index, SRI | $SRI=R_{\lambda1}/R_{\lambda2}$ | (Jordan, 1969;  Pearson & Miller, 1972) |
| normalized difference vegetation index, NDVI | $NDVI=(R_{\lambda1}-R_{\lambda2})/(R_{\lambda1}+R_{\lambda2})$ | (Rouse et al., 1974) |
| enhanced vegetation index, EVI2 | $EVI2=2.5\times{(R_{\lambda1}-R_{\lambda2})}/{(1+R_{\lambda1}+2.4R_{\lambda2})}$ | (Jiang et al., 2008) |
| optimized soil-adjusted vegetation index, OSAVI | $OSAVI=1.16\times(R_{\lambda1}-R_{\lambda2})/(R_{\lambda1}+R_{\lambda2}+0.16)$ | (Rondeaux et al., 1996) |
| soil-adjusted vegetation index, SAVI | $SAVI=1.5\times(R_{\lambda1}-R_{\lambda2})/(R_{\lambda1}+R_{\lambda2}+0.5)$ | (Huete, 1988) |
| difference vegetation index, DVI | $DVI=R_{\lambda1}-R_{\lambda2}$ | (Jordan, 1969) |
| renormalized difference vegetation index, RDVI | $RDVI=(R_{\lambda1}-R_{\lambda2})/\sqrt{(R_{\lambda1}+R_{\lambda2})}$ | (Roujean & Breon, 1995) |
| modified simple ratio, MSR | $MSR=\frac{{R_{\lambda1}}/{R_{\lambda2}}-1}{\left( \sqrt{{R_{\lambda1}}/{R_{\lambda2}}}-1 \right)}$ | (Chen, 1996) |
| modified soil-adjusted vegetation index, MSAVI | $MSAVI=0.5\times\left( 2R_{\lambda1}+1-\sqrt{\left( 2R_{\lambda1}+1 \right)^{2}-8\times\left( R_{\lambda1}-R_{\lambda2} \right)} \right)$ | (Qi et al., 1994) |
| NDSI | $\mathrm{NDSI}_{(D_{\lambda1}, D_{\lambda2})}=(D_{\lambda1}-D_{\lambda2})/(D_{\lambda1}+D_{\lambda2})$ | (Inoue et al., 2012) |
| RSI | $\mathrm{RSI}_{(D_{\lambda1}, D_{\lambda2})}=D_{\lambda1}/D_{\lambda2}$ | (Inoue et al., 2012;  Zarco-Tejada et al., 2009) |

Note: *R*, *G*, *B* denotes the radiometric normalized pixel values of each orthomosaic images’ red, green, and blue band, respectively. *R_λ1_* or *R_λ2_* represents the reflectance of a variable band in the spectral range of 600-1000 nm. *D_λ1_* and *D_λ2_* are the first derivative values at *λ1* and *λ2* nm over the whole spectra.

**Table A3.** The 68 common vegetation indices.

| **Vegetation indices** | | **Formula** | **References** |
| --- | --- | --- | --- |
| 1 | photochemical reflectance index, PRI | $\mathrm{PRI}_{570}=(R_{570}-R_{531})/(R_{570}+R_{531})$ | (Gamon et al., 1992) |
| 2 |  | $\mathrm{PRI}_{512}=(R_{512}-R_{531})/(R_{512}+R_{531})$ | (Hernández-Clemente et al., 2011) |
| 3 | simple ratio pigment index, SRPI | $SRPI=R_{430}/R_{680}$ | (Peñuelas et al., 1995) |
| 4 | normalized difference vegetation index, NDVI | $NDVI=(R_{800}-R_{670})/(R_{800}+R_{670})$ | (Rouse et al., 1974) |
| 5 | green normalized different vegetation index, GNDVI | $GNDVI=(R_{800}-R_{550})/(R_{800}+R_{550})$ | (Gitelson et al., 1996) |
| 6 | ND_705_ | $\mathrm{ND}_{705}=(R_{750}-R_{705})/(R_{750}+R_{705})$ | (Sims & Gamon, 2002) |
| 7 | modified red-edge normalized difference vegetation index，mND_705_ | $\mathrm{mND}_{705}=(R_{750}-R_{705})/(R_{750}+R_{705}-2R_{445})$ | (Sims & Gamon, 2002) |
| 8 | atmospheric resistant vegetation index, ARVI | $ARVI=(R_{800}-{(2R}_{680}-R_{450}))/(R_{800}+{(2R}_{680}-R_{450}))$ | (Kaufman & Tanré, 1996) |
| 9 | red edge vegetation stress index, RVSI | $RVSI=(R_{714}-R_{752})/2-R_{733}$ | (Merton, 1998) |
| 10 | renormalized difference vegetation index, RDVI | $RDVI=(R_{800}-R_{670})/\sqrt{(R_{800}+R_{670})}$ | (Roujean & Breon, 1995) |
| 11 | difference vegetation index, DVI | $DVI=R_{800}-R_{680}$ | (Jordan, 1969) |
| 12 | enhanced vegetation index, EVI | $EVI=2.5\times\frac{(R_{800}-R_{670})}{(1+R_{800}+6R_{670}-7R_{479})}$ | (Huete et al., 2002) |
| 13 | enhanced vegetation index, EVI2 | $EVI2=2.5\times\frac{(R_{800}-R_{660})}{(1+R_{800}+2.4R_{660})}$ | (Jiang et al., 2008) |
| 14 | chlorophyll absorption ratio index, CARI | $CARI=\left( R_{700}-R_{670} \right)-0.2\times(R_{700}-R_{550})$ | (Kim et al., 1994) |
| 15 | pigment-specific normalized difference, PSND | $\mathrm{PSND}_{a}=(R_{800}-R_{680})/(R_{800}+R_{680})$ | (Blackburn, 1998) |
| 16 |  | $\mathrm{PSND}_{b}=(R_{800}-R_{635})/(R_{800}+R_{635})$ |  |
| 17 |  | $\mathrm{PSND}_{c}=(R_{800}-R_{470})/(R_{800}+R_{470})$ |  |

| **Vegetation indices** | | **Formula** | **References** |
| --- | --- | --- | --- |
| 18 | Datt vegetation index | $\mathrm{Datt}=(R_{850}-R_{710})/(R_{850}-R_{680})$ | (Datt, 1999) |
| 19 | triangular vegetation index, TVI | $TVI=0.5\times\left[ 120\times\left( R_{750}-R_{550} \right)-200\times(R_{670}-R_{550}) \right]$ | (Broge & Leblanc, 2001) |
| 20 | modified triangular index, MTVI1 | $MTVI1=1.2\times\left[ 1.2\times\left( R_{800}-R_{550} \right)-2.5\times(R_{670}-R_{550}) \right]$ | (Haboudane et al., 2004) |
| 21 | Maccioni vegetation index | $\mathrm{Maccioni}=(R_{780}-R_{710})/(R_{780}-R_{680})$ | (Maccioni et al., 2001) |
| 22 | normalized pigment chlorophyll index, NPCI | $\mathrm{NPCI}=(R_{680}-R_{430})/(R_{680}+R_{430})$ | (Peñuelas et al., 1994) |
| 23 | structual independent pigment index, SIPI | $\mathrm{SIPI}=(R_{800}-R_{445})/(R_{800}+R_{680})$ | (Peñuelas et al., 1995) |
| 24 | optimized soil-adjusted vegetation index, OSAVI | $OSAVI=\frac{(1+0.16)\times(R_{800}-R_{670})}{(R_{800}-R_{670}+0.16)}$ | (Rondeaux et al., 1996) |
| 25 |  | $OSAVI2=\frac{(1+0.16)\times(R_{750}-R_{705})}{(R_{750}-R_{705}+0.16)}$ | (Wu et al., 2008) |
| 26 | modified soil-adjusted vegetation index, MSAVI | $MSAVI=0.5\times\left( 2R_{800}+1-\sqrt{\left( 2R_{800}+1 \right)^{2}-8\times\left( R_{800}-R_{670} \right)} \right)$ | (Qi et al., 1994) |
| 27 | simple ration, SR | $\mathrm{SR}=R_{800}/R_{670}$ | (Jordan, 1969;  Pearson & Miller, 1972) |
| 28 | modified simple ratio, MSR | $MSR=\frac{{R_{800}}/{R_{670}}-1}{\left( \sqrt{{R_{800}}/{R_{670}}}-1 \right)}$ | (Chen, 1996) |
| 29 | pigment-specific simple ratio, PSSR | $\mathrm{PSSR}_{a}=R_{800}/R_{680}$ | (Blackburn, 1998) |
| 30 |  | $\mathrm{PSSR}_{b}=R_{800}/R_{635}$ |  |
| 31 |  | $\mathrm{PSSR}_{c}=R_{800}/R_{470}$ |  |
| 32 | modified simple ratio index | $\mathrm{mSR}_{705}=(R_{750}-R_{445})/(R_{705}-R_{445})$ | (Sims & Gamon, 2002) |
| 33 | modified chlorophyll absorption in reflectance index, MCARI | $MCARI=\left[ \left( R_{700}-R_{670} \right)-0.2\times(R_{700}-R_{550}) \right]\times(R_{700}/R_{670})$ | (Daughtry et al., 2000) |
| 34 | transformed chlorophyll absorption in reflectance index, TCARI | $TCARI=3\times\left[ \left( R_{700}-R_{670} \right)-0.2(R_{700}-R_{550})\times(R_{700}/R_{670}) \right]$ | (Haboudane et al., 2002) |
| 35 | MERIS terrestrial chlorophyll index, MTCI | $\mathrm{MTCI}=(R_{754}-R_{709})/(R_{709}-R_{681})$ | (Dash & Curran, 2004) |
| 36 | nitrogen reflectance index, NRI | $NRI=(R_{570}-R_{670})/(R_{570}+R_{670})$ | (Diker & Bausch, 2003) |

| **Vegetation indices** | | **Formula** | **References** |
| --- | --- | --- | --- |
| 37 | MCARI/OSAVI | MCARI/OSAVI | (Daughtry et al., 2000) |
| 38 | TCARI/OSAVI | TCARI/OSAVI | (Haboudane et al., 2002) |
| 39 | plant senescence reflectance index, PSRI | $\mathrm{PSRI}=(R_{680}-R_{500})/R_{750}$ | (Sims & Gamon, 2002) |
| 40 | anthocyanin reflectance index, ARI | $ARI1=$1/$R_{550}-1/R_{700}$ | (Gitelson et al., 2002b) |
| 41 |  | $ARI2=R_{800}\times($1/$R_{550}-1/R_{700})$ |  |
| 42 | carotenoid reflectance index, CRI | $CRI1=$1/$R_{510}-1/R_{550}$ |  |
| 43 |  | $CRI2=$1/$R_{510}-1/R_{700}$ |  |
| 44 | Vogelmann indices, VOG | $VOG1=R_{740}/R_{720}$ | (Vogelmann et al., 1993) |
| 45 |  | $VOG2=(R_{734}-R_{747})/(R_{715}-R_{726})$ | (Zarco-Tejada et al., 2001) |
| 46 | Zarco and Miller, ZM | $ZM=R_{750}/R_{710}$ | (Zarco-Tejada et al., 2001) |
| 47 | water index, WI | $WI=R_{900}/R_{970}$ | (Peñuelas et al., 1995) |
| 48 | healthy index, HI | $HI=\frac{R_{534}-R_{698}}{R_{534}+R_{698}}-0.5R_{704}$ | (Mahlein et al., 2013) |
| 49 | greenness index | $R_{550}/R_{670}$ | (Carter, 1994) |
| 50 | blue/green pigment indices, BGI | $BGI1=R_{400}/R_{550}$ | (Zarco-Tejada et al., 2005) |
| 51 |  | $BGI2=R_{450}/R_{550}$ |  |
| 52 | blue/red pigment indices, BRI | $BRI1=R_{400}/R_{690}$ |  |
| 53 |  | $BRI2=R_{450}/R_{690}$ |  |
| 54 | Lichtenthaler index | $LIC3=R_{440}/R_{740}$ | (Lichtenthaler et al., 1996) |
| 55 | carotenoid concentration | $R_{520}/R_{500}$ | (Zarco-Tejada et al., 2012) |
| 56 |  | $R_{515}/R_{570}$ |  |
| 57 |  | $R_{515}/R_{670}$ |  |
| 58 | normalized difference red edge index, NDRE | $\mathrm{NDRE}=(R_{790}-R_{720})/(R_{790}+R_{720})$ | (Fitzgerald et al., 2010) |
| 59 | canopy chlorophyll content index, CCCI | $\mathrm{CCCI}=\frac{\left( \mathrm{NDRE}-\mathrm{NDRE}_{\mathrm{MIN}} \right)}{(\mathrm{NDRE}_{\mathrm{MAX}}+\mathrm{NDRE}_{\mathrm{MIN}})}$ | (Fitzgerald et al., 2010) |
| 60 | red-edge model, R-M | R-M$=R_{750}/R_{720}-1$ | (Gitelson et al., 2005) |
| 61 | green model, G-M | G-M$=R_{750}/R_{550}-1$ | (Gitelson et al., 2005) |
| 62 | red edge position, REP | $REP=700+40\times\frac{\left[ {(R}_{670}+R_{780})/2-R_{700} \right]}{{(R}_{740}+R_{700})}$ | (Guyot et al., 1988) |
| 63 | ratio spectral index, RSI | $\mathrm{RSI}_{(R_{\lambda1}, R_{\lambda2})}=R_{\lambda1}/R_{\lambda2}$ | (Zarco-Tejada et al., 2009; Inoue et al., 2012) |

| **Vegetation indices** | | **Formula** | **References** |
| --- | --- | --- | --- |
| 64 | normalized difference spectral index, NDSI | $\mathrm{NDSI}_{(R_{\lambda1}, R_{\lambda2})}=(R_{\lambda1}-R_{\lambda2})/(R_{\lambda1}+R_{\lambda2})$  $R_{\lambda1}$*>*$R_{\lambda2}$ | (Inoue et al., 2012) |
| 65 | difference spectral index, DSI | $\mathrm{DSI}_{(R_{\lambda1}, R_{\lambda2})}=R_{\lambda1}-R_{\lambda2}$ | (Jordan, 1969) |
| 66 | SAVI | $\mathrm{SAVI}_{(R_{\lambda1}, R_{\lambda2})}=1.5\times(R_{\lambda1}-R_{\lambda2})/(R_{\lambda1}+R_{\lambda2}+0.5)$ | (Huete, 1988) |
| 67 | NDSI | $\mathrm{NDSI}_{(D_{\lambda1}, D_{\lambda2})}=(D_{\lambda1}-D_{\lambda2})/(D_{\lambda1}+D_{\lambda2})$ | (Inoue et al., 2012) |
| 68 | RSI | $\mathrm{RSI}_{(D_{\lambda1}, D_{\lambda2})}=D_{\lambda1}/D_{\lambda2}$ | (Zarco-Tejada et al., 2009; Inoue et al., 2012) |

**Table A4.** Texture characteristic parameter calculation methods.

| No. | Texture characteristics | Formula | References |
| --- | --- | --- | --- |
| 1 | Average value | $M\text{ean}=\frac{1}{MN}\sum_{i=0}^{M-1} \sum_{j=0}^{N-1} f(i,j,d,\theta)$ | (Enu, 1985) |
| 2 | Variance | $Var=\sum_{i=0}^{M-1} \sum_{j=0}^{N-1} (i-\mu)^{2}f(i,j,d,\theta)$ | (Baraldi & Panniggiani, 1995) |
| 3 | Contrast ratio | $Con=\sum_{i=0}^{M-1} \sum_{j=0}^{N-1} (i-j)^{2}f(i,j,d,\theta)$ | (Hong, 2018) |
| 4 | Homogeneity | $Hom=\sum_{i=0}^{M-1} \sum_{j=0}^{N-1} f(i,j,d,\theta)/(i-j)^{2}$ | (Peña-Barragán et al., 2011) |
| 5 | Dissimilarity | $Dis=\sum_{i=0}^{M-1} \sum_{j=0}^{N-1} \left\vert i-j\right\vert f(i,j,d,\theta)$ | (Kim & Yeom, 2014) |
| 6 | Entropy | $Ent=-\sum_{i=0}^{M-1} \sum_{j=0}^{N-1} f(i,i)\lg f(i,j,d,\theta)$ | (Yang et al., 2020) |
| 7 | Angular second-order moment | $ASM=\sum_{i=0}^{M-1} \sum_{j=0}^{N-1} f(i,j,d,\theta)^{2}$ | (Treitz et al., 1996) |
| 8 | Relevance | $Cor=\sum_{i=0}^{M-1} \sum_{j=0}^{N-1} (i-\mu)(j-\mu)f(i,j,d,\theta)^{2}/\delta^{2}$ | (Guijarro et al., 2011) |
| 9 | Autocorrelation | $AutoCor=\sum_{i=0}^{M-1} \sum_{j=0}^{N-1} (ij)f(i,j,d,\theta)$ | (Jung et al., 2006) |

**Table A5.** Application of deep learning in crop diseases monitoring.

| Type | Crop | Diseases/ pests | Sensors | Deep learning method | Accuracy | References |
| --- | --- | --- | --- | --- | --- | --- |
| Detection | Pine Trees | Pine wilt disease | - | Faster R-CNN | 82.42% | (Xu et al., 2022) |
|  | Pine Trees | Dead trees with pine wilt disease | RGB | Faster R-CNN-based ResNet | 89.10% | (Deng et al., 2020) |
|  | Soybean | soybean leaf diseases | RGB | Inception-v3 with FT 75% | 99.04% | (Tetila et al., 2019) |
|  | Wheat | Wheat yellow rust disease | - | PSNet | 94% | (Pan et al., 2021) |
|  | Rapeseed material | Freezing injury | RGB | ResNet50 | 93.33% | (Li et al., 2022) |
|  | Moso bamboo forest | Pantana phyllostachysae chao | Multispectral | XGBoost | 86.47% | (Xu et al., 2022) |
|  | Maize | Maize drought | Hyperspectral | U-Net, SE-ResNeXt-50 | 91.66% | (Liu et al., 2020) |
|  | Yunnan Pine | Pine shoot beetle | Hyperspectral | - | 87.50% | (Liu et al., 2020) |
| Classification | Rice | rice pests | - | EfficientNet-B0 | 97.58% | (Yang et al., 2021) |
|  | Soybean | Soybean pests | RGB | SLIC, ResNet-50 | 93.82% | (Tetila et al., 2020) |
|  | Abies mariesii | Individual sick fir tree | RGB | ResNet | 98.09% | (Nguyen et al., 2021) |
|  | Plant leaf | Plant leaf disease | - | EfficientNet | 99.97% | (Atila et al., 2021) |

**References**

Abdulridha, J., Ampatzidis, Y., Ehsani, R., & de Castro, A. I. (2018). Evaluating the performance of spectral features and multivariate analysis tools to detect laurel wilt disease and nutritional deficiency in avocado. *Computers and Electronics in Agriculture*, *155*, 203-211. doi: 10.1016/j.compag.2018.10.016

Atila, Ü., Uçar, M., Akyol, K., & Uçar, E. (2021). Plant leaf disease classification using EfficientNet deep learning model. *Ecological Informatics*, *61*, 101182. doi: 10.1016/j.ecoinf.2020.101182

Baraldi, A., & Panniggiani, F. (1995). An investigation of the textural characteristics associated with gray level cooccurrence matrix statistical parameters. *IEEE transactions on geoscience and remote sensing*, *33*(2), 293-304. doi: 10.1109/TGRS.1995.8746010

Bendig, J., Yu, K., Aasen, H., Bolten, A., Bennertz, S., Broscheit, J., ... & Bareth, G. (2015). Combining UAV-based plant height from crop surface models, visible, and near infrared vegetation indices for biomass monitoring in barley. *International Journal of Applied Earth Observation and Geoinformation*, *39*, 79-87. doi: 10.1016/j.jag.2015.02.012

Blackburn, G. A. (1998). Spectral indices for estimating photosynthetic pigment concentrations: a test using senescent tree leaves. *International Journal of Remote Sensing*, *19*(4), 657-675. doi: 10.1080/014311698215919

Broge, N. H., & Leblanc, E. (2001). Comparing prediction power and stability of broadband and hyperspectral vegetation indices for estimation of green leaf area index and canopy chlorophyll density. *Remote sensing of environment*, *76*(2), 156-172. doi: [10.1016/S0034-4257(00)00197-8](https://doi.org/10.1016/S0034-4257(00)00197-8)

Carter, G. A. (1994). Ratios of leaf reflectances in narrow wavebands as indicators of plant stress. *Remote sensing*, *15*(3), 697-703. doi: 10.1080/01431169408954109

Chen, J. M. (1996). Evaluation of vegetation indices and a modified simple ratio for boreal applications. *Canadian Journal of Remote Sensing*, *22*(3), 229-242. doi: 10.1080/07038992.1996.10855178

Chouhan, S. S., Kaul, A., Singh, U. P., & Jain, S. (2018). Bacterial foraging optimization based radial basis function neural network (BRBFNN) for identification and classification of plant leaf diseases: An automatic approach towards plant pathology. *IEEE access*, *6*, 8852-8863. doi: 10.1109/ACCESS.2018.2800685

Dash, J., & Curran, P. J. (2004). The MERIS terrestrial chlorophyll index. doi: 10.1080/0143116042000274015

Datt, B. (1999). Remote sensing of water content in Eucalyptus leaves. *Australian Journal of botany*, *47*(6), 909-923. doi: 10.1071/BT98042

Daughtry, C. S., Walthall, C. L., Kim, M. S., De Colstoun, E. B., & McMurtrey Iii, J. E. (2000). Estimating corn leaf chlorophyll concentration from leaf and canopy reflectance. *Remote sensing of Environment*, *74*(2), 229-239. doi: [10.1016/S0034-4257(00)00113-9](https://doi.org/10.1016/S0034-4257(00)00113-9)

Deng, X., Tong, Z., Lan, Y., & Huang, Z. (2020). Detection and Location of Dead Trees with Pine Wilt Disease Based on Deep Learning and UAV Remote Sensing. *AgriEngineering*, *2*(2), 294-307. doi: 10.3390/agriengineering2020019

Dhingra, G., Kumar, V., & Joshi, H. D. (2019). A novel computer vision based neutrosophic approach for leaf disease identification and classification. *Measurement*, *135*, 782-794. doi: 10.1016/j.measurement.2018.12.027

Diker, K., & Bausch, W. C. (2003). Potential use of nitrogen reflectance index to estimate plant parameters and yield of maize. *Biosystems Engineering*, *85*(4), 437-447. doi: [10.1016/S1537-5110(03)00097-7](https://doi.org/10.1016/S1537-5110(03)00097-7)

Du, M., & Noguchi, N. (2017). Monitoring of wheat growth status and mapping of wheat yield’s within-field spatial variations using color images acquired from UAV-camera system. *Remote sensing*, *9*(3), 289. doi: 10.3390/rs9030289

Enu, E. (1985). Textural characteristics of the Nigerian tar sands. *Sedimentary geology*, *44*(1-2), 65-81. doi: 10.1016/0037-0738(85)90032-6

Fitzgerald, G., Rodriguez, D., & O’Leary, G. (2010). Measuring and predicting canopy nitrogen nutrition in wheat using a spectral index—The canopy chlorophyll content index (CCCI). *Field crops research*, *116*(3), 318-324. doi: 10.1016/j.fcr.2010.01.010

Fuentes, A. F., Yoon, S., Lee, J., & Park, D. S. (2018). High-performance deep neural network-based tomato plant diseases and pests diagnosis system with refinement filter bank. *Frontiers in plant science*, *9*, 1162. doi: 10.3389/fpls.2018.01162

Gamon, J. A., Penuelas, J., & Field, C. B. (1992). A narrow-waveband spectral index that tracks diurnal changes in photosynthetic efficiency. *Remote Sensing of environment*, *41*(1), 35-44. doi: 10.1016/0034-4257(92)90059-S

Gitelson, A. A., Kaufman, Y. J., Stark, R., & Rundquist, D. (2002a). Novel algorithms for remote estimation of vegetation fraction. *Remote sensing of Environment*, *80*(1), 76-87. doi: [10.1016/S0034-4257(01)00289-9](https://doi.org/10.1016/S0034-4257(01)00289-9)

Gitelson, A. A., Zur, Y., Chivkunova, O. B., & Merzlyak, M. N. (2002b). Assessing carotenoid content in plant leaves with reflectance spectroscopy. *Photochemistry and photobiology*, *75*(3), 272-281. doi: [10.1562/0031-8655(2002)0750272ACCIPL2.0.CO2](https://doi.org/10.1562/0031-8655(2002)0750272ACCIPL2.0.CO2)

Gitelson, A. A., Kaufman, Y. J., & Merzlyak, M. N. (1996). Use of a green channel in remote sensing of global vegetation from EOS-MODIS. *Remote Sensing of environment*, *58*(3), 289-298. doi: 10.1016/S0034-4257(96)00072-7

Gitelson, A. A., Viña, A., Ciganda, V., Rundquist, D. C., & Arkebauer, T. J. (2005). Remote estimation of canopy chlorophyll content in crops. *Geophysical research letters*, *32*(8). doi: 10.1029/2005GL022688

Guijarro, M., Pajares, G., Riomoros, I., Herrera, P., Burgos-Artizzu, X., & Ribeiro, A. (2011). Automatic segmentation of relevant textures in agricultural images. *Computers and Electronics in Agriculture*, *75*(1), 75-83. doi: 10.1016/j.compag.2010.09.013

Guerrero, J. M., Pajares, G., Montalvo, M., Romeo, J., & Guijarro, M. (2012). Support vector machines for crop/weeds identification in maize fields. *Expert Systems with Applications*, *39*(12), 11149-11155. doi: [10.1016/j.eswa.2012.03.040](https://doi.org/10.1016/j.eswa.2012.03.040)

Guyot, G., Baret, F., & Major, D. J. (1988). High spectral resolution: determination of spectral shifts between the red and near infrared. doi: 10.1029/JB095iB08p12653

Haboudane, D., Miller, J. R., Tremblay, N., Zarco-Tejada, P. J., & Dextraze, L. (2002). Integrated narrow-band vegetation indices for prediction of crop chlorophyll content for application to precision agriculture. *Remote sensing of environment*, *81*(2-3), 416-426. doi: [10.1016/S0034-4257(02)00018-4](https://doi.org/10.1016/S0034-4257(02)00018-4)

Haboudane, D., Miller, J. R., Pattey, E., Zarco-Tejada, P. J., & Strachan, I. B. (2004). Hyperspectral vegetation indices and novel algorithms for predicting green LAI of crop canopies: Modeling and validation in the context of precision agriculture. *Remote sensing of environment*, *90*(3), 337-352. doi: [10.1016/j.rse.2003.12.013](https://doi.org/10.1016/j.rse.2003.12.013)

Hague, T., Tillett, N. D., & Wheeler, H. J. P. A. (2006). Automated crop and weed monitoring in widely spaced cereals. *Precision Agriculture*, *7*, 21-32. doi: 10.1007/s11119-005-6787-1

Hernández-Clemente, R., Navarro-Cerrillo, R. M., Suárez, L., Morales, F., & Zarco-Tejada, P. J. (2011). Assessing structural effects on PRI for stress detection in conifer forests. *Remote Sensing of environment*, *115*(9), 2360-2375. doi: 10.1016/j.rse.2011.04.036

Hong, J.-Y. (2018). A study on characteristics related to texture, colour temperature and contrast ratio to improve the depth of stereoscopic images. *The Journal of The Institute of Internet, Broadcasting and Communication*, *18*(4), 37-42. doi: 10.7236/JIIBC.2018.18.4.37

Huete, A. R. (1988). A soil-adjusted vegetation index (SAVI). *Remote sensing of environment*, *25*(3), 295-309. doi: [10.1016/0034-4257(88)90106-X](https://doi.org/10.1016/0034-4257(88)90106-X)

Huete, A., Didan, K., Miura, T., Rodriguez, E. P., Gao, X., & Ferreira, L. G. (2002). Overview of the radiometric and biophysical performance of the MODIS vegetation indices. *Remote Sensing of environment*, *83*(1-2), 195-213. doi: 10.1016/S0034-4257(02)00096-2

Hunt Jr, E. R., Doraiswamy, P. C., McMurtrey, J. E., Daughtry, C. S., Perry, E. M., & Akhmedov, B. (2013). A visible band index for remote sensing leaf chlorophyll content at the canopy scale. *International journal of applied earth observation and Geoinformation*, *21*, 103-112. [doi: 10.1016/j.jag.2012.07.020](https://doi.org/10.1016/j.jag.2012.07.020)

Inoue, Y., Sakaiya, E., Zhu, Y., & Takahashi, W. (2012). Diagnostic mapping of canopy nitrogen content in rice based on hyperspectral measurements. *Remote Sensing of Environment*, *126*, 210-221. doi: [10.1016/j.rse.2012.08.026](https://doi.org/10.1016/j.rse.2012.08.026)

Jiang, Z., Huete, A. R., Didan, K., & Miura, T. (2008). Development of a two-band enhanced vegetation index without a blue band. *Remote Sensing of environment*, *112*(10), 3833-3845. doi: 10.1016/j.rse.2008.06.006

Jordan, C. F. (1969). Derivation of leaf‐area index from quality of light on the forest floor. *Ecology*, *50*(4), 663-666. doi: 10.2307/1936256

Jung, W., Kitchen, N., Sudduth, K., & Anderson, S. (2006). Spatial characteristics of claypan soil properties in an agricultural field. *Soil Science Society of America Journal*, *70*(4), 1387-1397. doi: 10.2136/sssaj2005.0273

Kaufman, Y. J., & Tanre, D. (1996). Strategy for direct and indirect methods for correcting the aerosol effect on remote sensing: from AVHRR to EOS-MODIS. *Remote Sensing of environment*, *55*(1), 65-79. doi: 10.1016/0034-4257(95)00193-X

Kim, H.-O., & Yeom, J.-M. (2014). Effect of red-edge and texture features for object-based paddy rice crop classification using RapidEye multi-spectral satellite image data. *International Journal of Remote Sensing*, *35*(19), 7046-7068. doi: 10.1080/01431161.2014.965285

Kim, M. S., Daughtry, C., Chappelle, E., McMurtrey, J., & Walthall, C. (1994). The use of high spectral resolution bands for estimating absorbed photosynthetically active radiation (A par). In *CNES, proceedings of 6th international symposium on physical measurements and signatures in remote sensing,* No. GSFC-E-DAA-TN72921.

Kouadio, L., Deo, R. C., Byrareddy, V., Adamowski, J. F., & Mushtaq, S. (2018). Artificial intelligence approach for the prediction of Robusta coffee yield using soil fertility properties. *Computers and Electronics in Agriculture*, *155*, 324-338. doi: 10.1016/j.compag.2018.10.014

Li, L., Qiao, J., Yao, J., Li, J., & Li, L. (2022). Automatic freezing-tolerant rapeseed material recognition using UAV images and deep learning. *Plant methods*, *18*(1), 1-13. doi: 10.1186/s13007-022-00838-6

Lichtenthaler, H. K., Lang, M., Sowinska, M., Heisel, F., & Miehe, J. A. (1996). Detection of vegetation stress via a new high resolution fluorescence imaging system. *Journal of plant physiology*, *148*(5), 599-612. doi: 10.1016/S0176-1617(96)80081-2

Liu, C., Li, H., Su, A., Chen, S., & Li, W. (2020). Identification and grading of maize drought on rgb images of uav based on improved u-net. *IEEE geoscience and remote sensing letters*, *18*(2), 198-202. doi: 10.1109/LGRS.2020.2972313

Maccioni, A., Agati, G., & Mazzinghi, P. (2001). New vegetation indices for remote measurement of chlorophylls based on leaf directional reflectance spectra. *Journal of Photochemistry and Photobiology B: Biology*, *61*(1-2), 52-61. doi: [10.1016/S1011-1344(01)00145-2](https://doi.org/10.1016/S1011-1344(01)00145-2)

Mahlein, A. K., Rumpf, T., Welke, P., Dehne, H. W., Plümer, L., Steiner, U., & Oerke, E. C. (2013). Development of spectral indices for detecting and identifying plant diseases. *Remote Sensing of Environment*, *128*, 21-30. doi: [10.1016/j.rse.2012.09.019](https://doi.org/10.1016/j.rse.2012.09.019)

Merton, R. (1998). Monitoring community hysteresis using spectral shift analysis and the red-edge vegetation stress index. In *Proceedings of the Seventh Annual JPL Airborne Earth Science Workshop* (pp. 12-16). JPL Pasadena, CA, USA.

Meyer, G. E., & Neto, J. C. (2008). Verification of color vegetation indices for automated crop imaging applications. *Computers and electronics in agriculture*, *63*(2), 282-293. doi: [10.1016/j.compag.2008.03.009](https://doi.org/10.1016/j.compag.2008.03.009)

Motohka, T., Nasahara, K. N., Oguma, H., & Tsuchida, S. (2010). Applicability of green-red vegetation index for remote sensing of vegetation phenology. *Remote Sensing*, *2*(10), 2369-2387. doi: 10.3390/rs2102369

Nguyen, H. T., Lopez Caceres, M. L., Moritake, K., Kentsch, S., Shu, H., & Diez, Y. (2021). Individual sick fir tree (Abies mariesii) identification in insect infested forests by means of UAV images and deep learning. *Remote Sensing*, *13*(2), 260. doi: 10.3390/rs13020260

Pan, Q., Gao, M., Wu, P., Yan, J., & Li, S. (2021). A deep-learning-based approach for wheat yellow rust disease recognition from unmanned aerial vehicle images. *Sensors*, *21*(19), 6540. doi: 10.3390/s21196540

Pearson, R. L., & Miller, L. D. (1972). Remote mapping of standing crop biomass for estimation of the productivity of the shortgrass prairie, Pawnee National Grasslands, Colorado. doi: 10.5555/19740715986

Peña-Barragán, J. M., Ngugi, M. K., Plant, R. E., & Six, J. (2011). Object-based crop identification using multiple vegetation indices, textural features and crop phenology. *Remote Sensing of Environment*, *115*(6), 1301-1316. doi: 10.1016/j.rse.2011.01.009

Peñuelas, J., Gamon, J., Fredeen, A., Merino, J., & Field, C. (1994). Reflectance indices associated with physiological changes in nitrogen-and water-limited sunflower leaves. *Remote Sensing of environment*, *48*(2), 135-146. doi: 10.1016/0034-4257(94)90136-8

Peñuelas, J., Filella, I., & Gamon, J. A. (1995). Assessment of photosynthetic radiation‐use efficiency with spectral reflectance. *New Phytologist*, *131*(3), 291-296. doi: 10.1111/j.1469-8137.1995.tb03064.x

Pineda, M., Pérez-Bueno, M. L., & Barón, M. (2018). Detection of bacterial infection in melon plants by classification methods based on imaging data. *Frontiers in plant science*, *9*, 164. doi: 10.3389/fpls.2018.00164

Qi, J., Chehbouni, A., Huete, A. R., Kerr, Y. H., & Sorooshian, S. (1994). A modified soil adjusted vegetation index. *Remote sensing of environment*, *48*(2), 119-126. doi: 10.1016/0034-4257(94)90134-1

Rondeaux, G., Steven, M., & Baret, F. (1996). Optimization of soil-adjusted vegetation indices. *Remote sensing of environment*, *55*(2), 95-107. doi: [10.1016/0034-4257(95)00186-7](https://doi.org/10.1016/0034-4257(95)00186-7)

Roujean, J.-L., & Breon, F.-M. (1995). Estimating PAR absorbed by vegetation from bidirectional reflectance measurements. *Remote Sensing of environment*, *51*(3), 375-384. doi: 10.1016/0034-4257(94)00114-3

Rouse, J. W., Haas, R. H., Schell, J. A., & Deering, D. W. (1974). Monitoring vegetation systems in the Great Plains with ERTS. *NASA Spec. Publ*, *351*(1), 309.

Sims, D. A., & Gamon, J. A. (2002). Relationships between leaf pigment content and spectral reflectance across a wide range of species, leaf structures and developmental stages. *Remote Sensing of environment*, *81*(2-3), 337-354. doi: 10.1016/S0034-4257(02)00010-X

Tetila, E. C., Machado, B. B., Astolfi, G., de Souza Belete, N. A., Amorim, W. P., Roel, A. R., & Pistori, H. (2020). Detection and classification of soybean pests using deep learning with UAV images. *Computers and Electronics in Agriculture*, *179*, 105836. doi: 10.1016/j.compag.2020.105836

Tetila, E. C., Machado, B. B., Menezes, G. K., Oliveira, A. d. S., Alvarez, M., Amorim, W. P., Belete, N. A. D. S., Da Silva, G. G., & Pistori, H. (2019). Automatic recognition of soybean leaf diseases using UAV images and deep convolutional neural networks. *IEEE geoscience and remote sensing letters*, *17*(5), 903-907. doi: 10.1109/LGRS.2019.2932385

Torres-Sánchez, J., Peña, J. M., de Castro, A. I., & López-Granados, F. (2014). Multi-temporal mapping of the vegetation fraction in early-season wheat fields using images from UAV. *Computers and Electronics in Agriculture*, *103*, 104-113. doi: 10.1016/j.compag.2014.02.009

Treitz, P. M., Howarth, P., & Soulis, E. (1996). Textural processing of multi-polarization SAR for agricultural crop classification. In *IGARSS'96. 1996 International Geoscience and Remote Sensing Symposium* (Vol. 4, pp. 1986-1988). IEEE. doi: 10.1109/IGARSS.1996.516864

Verrelst, J., Schaepman, M. E., Koetz, B., & Kneubühler, M. (2008). Angular sensitivity analysis of vegetation indices derived from CHRIS/PROBA data. *Remote Sensing of Environment*, *112*(5), 2341-2353. doi: 10.1016/j.rse.2007.11.001

Vogelmann, J. E., Rock, B. N., & Moss, D. M. (1993). Red edge spectral measurements from sugar maple leaves. *TitleREMOTE SENSING*, *14*(8), 1563-1575. doi: 10.1080/01431169308953986

Woebbecke, D. M., Meyer, G. E., Von Bargen, K., & Mortensen, D. A. (1995). Color indices for weed identification under various soil, residue, and lighting conditions. *Transactions of the ASAE*, *38*(1), 259-269. doi: 10.13031/2013.27838

Wu, C., Niu, Z., Tang, Q., & Huang, W. (2008). Estimating chlorophyll content from hyperspectral vegetation indices: Modeling and validation. *Agricultural and forest meteorology*, *148*(8-9), 1230-1241. doi: [10.1016/j.agrformet.2008.03.005](https://doi.org/10.1016/j.agrformet.2008.03.005)

Xu, Z., Zhang, Q., Xiang, S., Li, Y., Huang, X., Zhang, Y., Zhou, X., Li, Z., Yao, X., & Li, Q. (2022). Monitoring the severity of Pantana phyllostachysae Chao infestation in Moso bamboo forests based on UAV multi-spectral remote sensing feature selection. *Forests*, *13*(3), 418. doi: 10.3390/f13030418

Yang, G., Chen, G., Li, C., Fu, J., Guo, Y., & Liang, H. (2021). Convolutional rebalancing network for the classification of large imbalanced rice pest and disease datasets in the field. *Frontiers in plant science*, *12*, 671134. doi: 10.3389/fpls.2021.671134

Yang, N., Yu, J., Wang, A., Tang, J., Zhang, R., Xie, L., Shu, F., & Kwabena, O. P. (2020). A rapid rice blast detection and identification method based on crop disease spores' diffraction fingerprint texture. *Journal of the Science of Food and Agriculture*, *100*(9), 3608-3621. doi: 10.1002/jsfa.10383

Zarco-Tejada, P. J., Miller, J. R., Noland, T. L., Mohammed, G. H., & Sampson, P. H. (2001). Scaling-up and model inversion methods with narrowband optical indices for chlorophyll content estimation in closed forest canopies with hyperspectral data. *IEEE Transactions on Geoscience and Remote Sensing*, *39*(7), 1491-1507. doi: [10.1109/36.934080](https://doi.org/10.1109/36.934080)

Zarco-Tejada, P. J., Berjón, A., Lopez-Lozano, R., Miller, J. R., Martín, P., Cachorro, V., ... & De Frutos, A. (2005). Assessing vineyard condition with hyperspectral indices: Leaf and canopy reflectance simulation in a row-structured discontinuous canopy. *Remote Sensing of Environment*, *99*(3), 271-287. doi: 10.1016/j.rse.2005.09.002

Zarco-Tejada, P. J., Berni, J. A., Suárez, L., Sepulcre-Cantó, G., Morales, F., & Miller, J. R. (2009). Imaging chlorophyll fluorescence with an airborne narrow-band multispectral camera for vegetation stress detection. *Remote Sensing of Environment*, *113*(6), 1262-1275. doi: [10.1016/j.rse.2009.02.016](https://doi.org/10.1016/j.rse.2009.02.016)

Zarco-Tejada, P. J., González-Dugo, V., & Berni, J. A. (2012). Fluorescence, temperature and narrow-band indices acquired from a UAV platform for water stress detection using a micro-hyperspectral imager and a thermal camera. *Remote sensing of environment*, *117*, 322-337. doi: 10.1016/j.rse.2011.10.007
